# Shh agonist enhances maturation in homotypic Lgr5-positive inner ear organoids

**DOI:** 10.7150/thno.107345

**Published:** 2025-04-13

**Authors:** Nathaniel T Carpena, So-Young Chang, Seyoung Mun, Kyung Wook Kim, Hyun C Yoon, Phil-Sang Chung, Ji-Hun Mo, Jin-Chul Ahn, Ji On Park, Kyudong Han, Ji Eun Choi, Jae Yun Jung, Min Young Lee

**Affiliations:** 1 Department of Medical Laser, Graduate School of Medicine, Dankook University, Cheonan 31116, Republic of Korea.; 2 Beckman Laser Institute Korea, Dankook University, Cheonan 31116, Republic of Korea.; 3 Department of Cosmedical & Materials, Dankook University, Cheonan 31116, Republic of Korea.; 4 Department of Orthopaedic Surgery, Dankook University Hospital, Dankook University College of Medicine, Cheonan, 31116, Korea.; 5 Department of Molecular Science & Technology, Ajou University, Suwon, 443749, Republic of Korea.; 6 Department of Otolaryngology-Head & Neck Surgery, College of Medicine, Dankook University, Cheonan 31116, Republic of Korea.; 7 Medical Laser Research Center, Dankook University, Cheonan 31116, Republic of Korea.; 8 Department of Microbiology, College of Science & Technology, Dankook University, Cheonan 31116, Republic of Korea.

**Keywords:** inner ear organoid, Lgr5, sonic hedgehog, RNA sequencing, microelectrode array

## Abstract

**Background:** The regeneration of functional hair cells (HCs) remains a critical challenge in addressing sensorineural hearing loss. This study aimed to investigate the molecular and functional mechanisms driving stereocilia maturation within inner ear organoids (IEO) derived from homogenic Lgr5-positive progenitor cells (LPCs) and to compare outcomes with traditional heterotypic cultures.

**Methods:** Mouse cochlear LPCs were isolated via magnetic-activated cell sorting (MACS) to establish homotypic cultures, ensuring purity and eliminating the heterotypic influences present in traditional manual isolation (MI) methods. Differentiation into HCs was induced through Wnt and Notch signaling modulation. Transcriptomic profiling using bulk and single-cell RNA sequencing (scRNA-seq) identified gene expression changes linked to stereocilia development. A Sonic Hedgehog (Shh) agonist was applied to enhance structural maturation of HCs. Functional assessment included electron microscopy, FM1-43 uptake assays, and microelectrode array recordings in assembloids of IEO with primary spiral ganglion neurons (SGN) co-cultures.

**Results:** While homotypic LPC-derived IEOs successfully differentiated into HC-like cells, initial morphological assessment revealed immature stereocilia structures. Bulk RNA-seq analysis highlighted a downregulation of morphogenesis-related genes in these organoids. The application of a Shh agonist, acting as a key morphogen, promoted stereocilia development, as evidenced by enhanced ultrastructural features and increased expression of cuticular plate-associated genes (*Pls1, Lmo7* and* Lrba)*. Single-cell RNA sequencing (scRNA-seq) further identified distinct cell clusters, which exhibited robust expression of stereocilia-related genes (*Espn, Lhfpl5, Loxhd1* and* Tmc1)*, indicative of advanced HC maturation. Electrophysiological assessments of IEO-SGN assembloids using microelectrode arrays confirmed functional mechanoelectrical transduction between cells.

**Conclusion:** This integrated approach elucidates critical pathways and cellular dynamics underpinning stereocilia maturation and functional HC development in EIOs. These findings provide new insights into the molecular regulation of HC maturation and support the utility of Shh-modulated IEOs as a promising platform for inner ear regeneration and therapeutic development for inner ear regenerative therapies.

## Introduction

Sensorineural hearing loss, primarily caused by irreversible damage to cochlear hair cells (HCs), remains a significant clinical challenge 1. HCs are specialized sensory cells that convert mechanical stimuli into electrical signals through mechanoelectrical transduction, a process facilitated by their apical stereocilia bundles 2. Deflection of these stereocilia by endolymph flow leads to ion channel activation, generating electrical signals that are transmitted to the brain 3, 4.

Efforts in regenerative medicine have focused on restoring lost HCs to address sensorineural hearing loss. A promising avenue in this field involves the use of organoid cultures derived from progenitor or stem cells, which can self-organize into three-dimensional (3D) structures that recapitulate key aspects of native tissue architecture and function 5-7. Inner ear organoids (IEOs) have been successfully generated from embryonic stem cells (ESCs) using stepwise differentiation protocols that mimic developmental signaling pathways 8-11. These ESC-derived IEOs have provided valuable models for studying inner ear development and disease 12, 13. However, ESC-derived IEOs often result in heterogeneous populations, making it challenging to focus specifically on cochlear hair cell development 10, 14. To overcome this limitation, research has shifted towards using progenitor cells, which serve as a robust and more targeted source for HC regeneration. Organoids derived from progenitor cells offer several advantages over embryonic cultures, including reduced cellular heterogeneity, enhanced reproducibility, and more controlled differentiation trajectories focused specifically on generating cochlear HCs 15-17.

Leucine-rich repeat-containing G-protein-coupled receptor 5 (Lgr5) is a well-established marker of cochlear sensory progenitors and plays a vital role in HC development and regeneration 18-20. Lgr5-positive cells (LPC) exhibit stem cell-like properties and have been shown to contribute to HC regeneration in the cochlea, making them an attractive target for organoid development. Traditional methods for LPC isolation, such as manual isolation (MI) via stripping of the sensory epithelia, often yield heterotypic cultures that include various supporting cells and other non-progenitor cell types 18, 21-23. This cellular heterogeneity complicates the study of specific cell-cell interactions that influence HC differentiation 24. To address this, Fluorescence-Activated Cell Sorting (FACS) has been employed to isolate pure populations of LPCs, enabling the generation of homotypic cochlear organoids. However, a major challenge in generating functionally mature HCs in homotypic organoids is promoting proper stereociliary maturation 25, 26. Stereocilia are essential for HC function, and their defective development impairs mechanoelectrical transduction. Understanding the molecular pathways that regulate stereociliary formation and maturation is therefore crucial for advancing inner ear regenerative therapies.

Transcriptomic approaches, including bulk RNA sequencing (RNA-seq) and single-cell RNA sequencing (scRNA-seq), have been instrumental in unraveling the gene expression landscapes that drive HC differentiation and stereociliary maturation 27, 28. These techniques allow for the identification of key signaling pathways and molecular markers associated with HC development.

In this study, we compared the differentiation of homotypic and heterotypic isolated progenitor cell-derived organoids to determine the roles of cellular interactions during the differentiation process. We utilized a combination of transcriptomic and functional analyses to identify signaling pathways that promote stereocilia development. Our findings highlight the pivotal role of Sonic hedgehog (Shh) signaling combined with Wnt inhibition in enhancing stereocilia maturation. We further validated these findings using ultrastructural imaging and electrophysiological assessments, confirming the generation of functionally mature HCs.

This integrated approach provides new insights into the molecular mechanisms underlying stereociliary maturation and offers a promising strategy for advancing inner ear regenerative therapies, with potential implications for treating sensorineural hearing loss.

## Results

### Lgr5-positive cell isolation and spheroid formation

**Figure 1:**
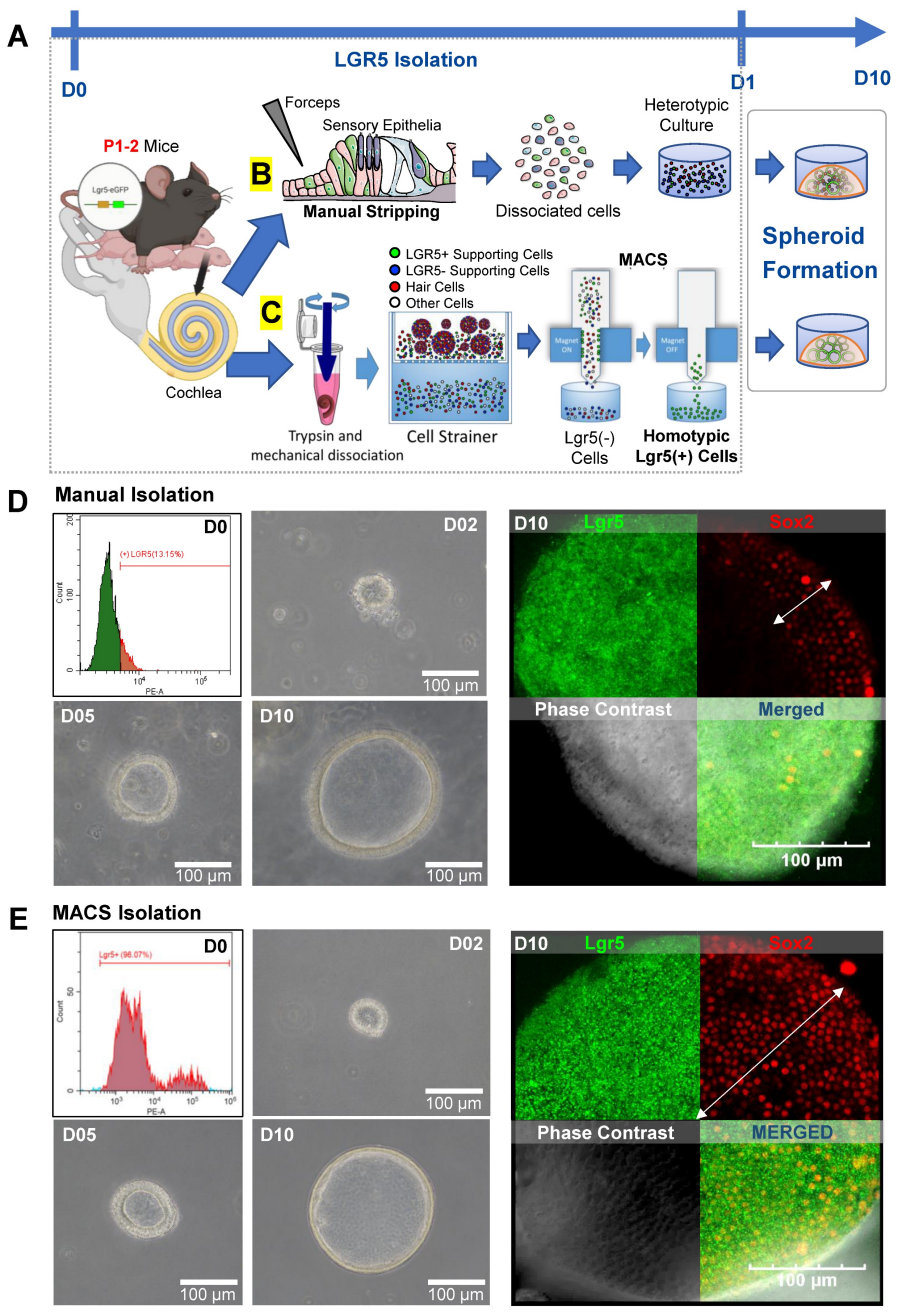
** Isolation of Lgr5 positive cells.** This figure shows images and illustrations of MI and MACS of LPCs. **(A)** On P1 or P2, cochleae were harvested from either transgenic Lgr5-GFP or wild-type mouse pups. Spheroid formation in cochlear tissue was then achieved in a 3D culture containing 100% Matrigel for 10 days. **(B)** For MI, the sensory epithelia were microdissected and manually stripped using forceps to isolate the HCs and support cells from the mesenchyme. All of the stripped cells were used to form spheroids of heterotypic culture. **(C)** Conversely, for MACS isolation, whole cochleae were enzymatically digested, mechanically dissociated via trituration, and strained into single cells. Dissociated cells were labeled with anti-Lgr5 magnetic microbeads, and the solution was passed through a magnetic field to remove the unlabeled cells. Upon removal from the magnetic field, the LPCs were collected and placed in a Matrigel 3D culture for 10 days to enable the formation of spheroids in homotypic cell culture. **(D)** FACS was conducted to measure the proportion of isolated LPCs. In the MI group, only 13.15% of the cells were Lgr5-positive. Brightfield microscopy confirmed the formation of spheroids within 10 days in vitro. IF staining showed that spheroids in the MI group were mostly positive for Lgr5, while Sox2 expression was minimal and dispersed (white arrow). **(E)** The LPCs in the MACS group had a purity of 96.07% and developed into spheroids with a similar morphology to those in the MI group. Immunostaining also showed that the spheroids in the MACS group were 97% positive for Sox2, while 78% were double positive for Lgr5 and Sox2. FACS; Fluorescence-activated cell sorting, GFP; Green fluorescent protein, HCs; Hair cells, IF; Immunofluorescence, Lgr5; Leucine-rich repeat-containing G-protein-coupled receptor 5, LPCs; Lgr5-positive cells, MACS; Magnetic-activated cell sorting, MI; Manual isolation, P; Postnatal day, 3D; Three-dimensional.

As mentioned above, a subset of the supporting cells in the cochlear sensory epithelia are crucial for regenerating and maintaining HCs. These cells are positive for the Lgr5 marker, which indicates that they play a role as progenitor cells in the inner ear. Using transgenic mouse pups that expressed a fluorescent protein in LPCs, we tested two isolation protocols for obtaining a population of these cells to use in the generation of spheroids **(Figure 1A)**. One isolation protocol involved the MI or stripping of the cochlear sensory epithelia using forceps to obtain a single-cell suspension **(Figure 1B, Figure S1)**. The resulting cell suspension was a heterotypic culture of Lgr5-positive supporting cells, along with Lgr5-negative supporting cells and HCs. The other isolation protocol utilized magnetic-activated cell sorting (MACS), where whole cochleae were completely dissociated, and LPCs were sorted based on their affinity to magnetic microbeads conjugated with an Lgr5 antibody **(Figure 1C, Figure S2)**. The resulting cell population obtained using the MACS method was a homotypic culture of pure LPCs.

FACS showed that of the cells obtained via MI, only 13.15% were Lgr5-positive **(Figure 1D)**. In contrast, 96.07% of the cells isolated via MACS were LPCs **(Figure 1E)**. The LPCs isolated from each group were expanded to generate spheroids via cell division for 10 days *in vitro*. At 7 days after isolation, the cluster of LPCs showed Lgr5, PAX2, and PAX8 expression, along with partial Oct4 and Sox2 expression **(Figure S3)**. The spheroids generated using the two methods were comparable in size and morphology, as seen from images of the spheroids **(Figures S3-S5)**. Fluorescence staining of the spheroids confirmed the presence of Lgr5-GFP-positive cells in both the MI and MACS groups **(Figure 1D-E)**. However, the MI group showed scant and dispersed Sox2 expression (white arrow) compared with the MACS group, which showed more consistent Sox2 expression. In the MI and MACS groups, 97% and 78% of the cells were double positive for Lgr5 and Sox2 expression, respectively. This result can be attributed to the presence of other cells within the heterotypic culture in the MI group that influenced the expression of Sox2 in the LPCs.

### LPCs differentiated into HCs

**Figure 2:**
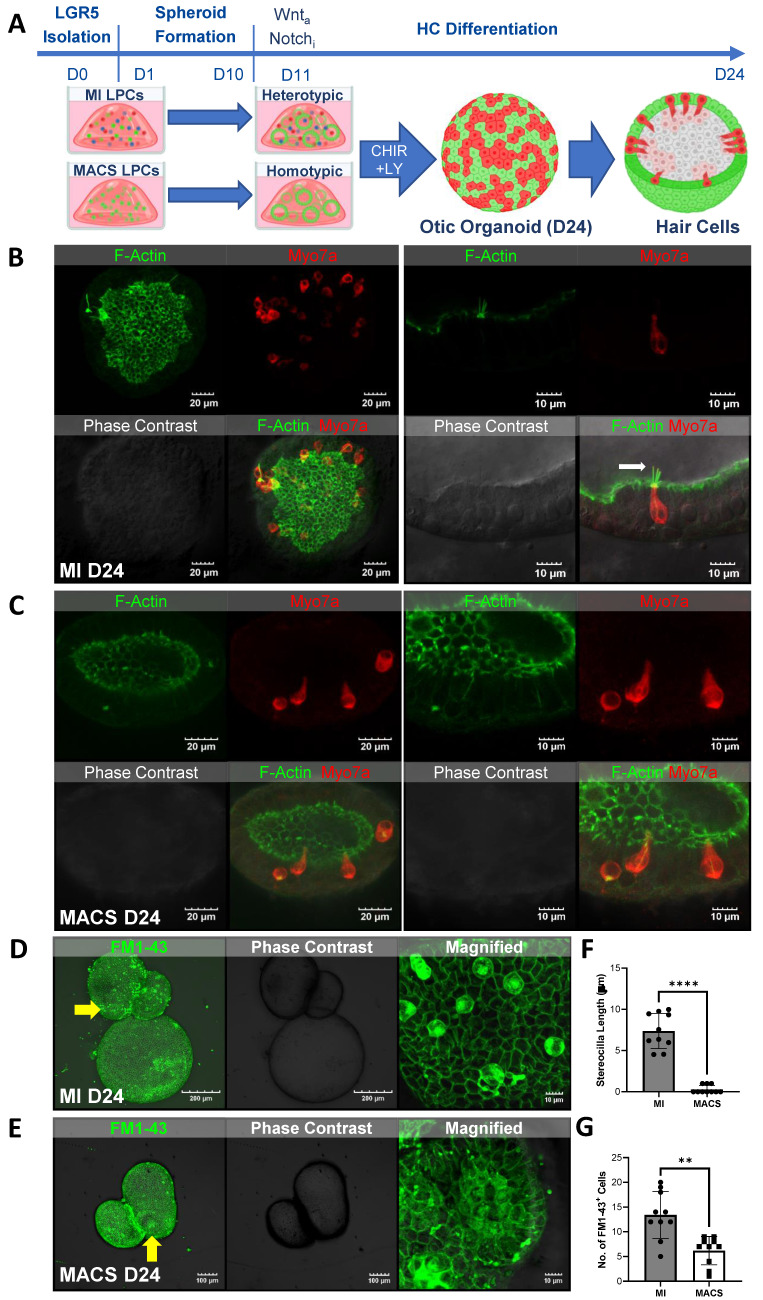
** LPCs differentiated into HCs. (A)** The illustration shows the timeline of LPC isolation via MI or MACS for in vitro formation of 3D structures or spheroids within 10 days. HC differentiation was performed immediately and lasted 14 days via the addition of a Wnt agonist and Notch inhibitor. **(B)** Otic organoids from the MI group that were sampled at the end of D24 had differentiated into HCs. Representative images of fully differentiated organoids show many Myo7a-positive cells with visible F-actin-rich areas. The figure shows a highly magnified image (right) of a Myo7a cell with a clear cilia-like structure and Myo7a expression at the tip (white arrow). **(C)** Immunofluorescence imaging of MACS spheroids revealed Myo7a-positive HCs, although F-actin staining did not indicate distinct stereocilia bundles at D24. **(D)** FM1-43 staining reflects MET, which is exhibited by mature HCs with functional stereocilia. MI-cultured organoids showed clear internalization, suggesting that MET channels were present and presumably functional in the differentiated putative HCs. **(E)** FM1-43 staining of MACS organoids elicited mostly extracellular staining, meaning that the cells were not successful in absorbing the dye and thus that no MET channels were present**. (F-G)** The stereocilia count and numbers of FM1-43-positive cells revealed a lack of HC functionality in the MACS spheroids, indicating that they were in an "immature" state. **p < 0.01; ****p < 0.0001. D; Day, FM1-43; N-(3-triethylammoniumpropyl)-4-(4-(dibutylamino)styryl) pyridinium dibromide, HCs; Hair cells, IF; Immunofluorescence, Lgr5; Leucine-rich repeat-containing G-protein-coupled receptor 5, LPCs; Lgr5-positive cells, MACS; Magnetic-activated cell sorting, MET; Mechanotransduction, MI; manual isolation, Myo7a; Myosin 7a, 3D; three-dimensional.

HC differentiation was induced in the MI and MACS spheroids using a small-molecule approach. Specifically, we targeted Wnt signaling via the GSK3β inhibitor CHIR99021 and induced transcriptional activation via the γ-secretase inhibitor LY411575 to concurrently inhibit Notch **(Figure 2A)**. Epifluorescence analysis performed 10 days after differentiation indicated that all the organoids were expressing Lgr5. Some organoids were also expressing Pax2 or Myo7a **(Figure S4)**. When comparing the MACS and MI cultures via polymerase chain reaction (PCR) at similar time points, the MI group showed higher expression of laminin, Pax8, and Atoh1, while the MACS cultures showed higher expression of Pax2 **(Figure S6)**. After 2 weeks of differentiation, the LPCs yielded HCs. These were assessed for HC markers, including Myo7a and F-actin, via immunostaining. HCs from the MI group that were positive for Myo7a (red) staining also developed distinct F-actin (green)-expressing protrusions similar to the native stereocilia of HCs **(Figure 2B)**. Lgr5 expression was decreased compared with the early differentiation stage **(Figures S4)**. Images taken at a higher magnification showed Myo7a-positive cells with a clearly visible cilia-like structure exhibiting Myo7a expression at the tip (white arrow).

The otic organoids in the homotypic MACS culture were able to successfully differentiate into HCs. Specifically, immunofluorescence imaging showed a similar number of Myo7a-positive HCs between the MACS and MI groups **(Figure S5A)**. However, highly magnified images showed significantly fewer F-actin stereocilia bundles on D24 **(Figure 2C, Figure S5)**. Furthermore, Lgr5 expression varied within single organoids. Espin (stereocilia marker) expression was very rare **(Figure S5B)**.

Small styryl dyes were used to assess the functional characteristics of the HCs in the cochlea. N-(3-triethylammoniumpropyl)-4-(4-(dibutylamino)styryl) pyridinium dibromide (FM1-43) uptake assays were performed on the spheroids generated via both the MI and MACS methods. The results showed that the HCs in the spheroids generated from the MI method exhibited robust FM1-43 uptake **(Figure 2D, F)**, indicating functional integrity and activity. However, the HCs in the spheroids generated from the MACS method showed minimal FM1-43 uptake **(Figure 2E, G)**, suggesting a potential lack of functional characteristics despite their HC phenotype. Thus, these HCs were considered "immature". These findings suggest that the heterotypic culture of Lgr5-positive supporting cells in the MI group contributed to the functional characteristics of the HCs.

### Exploring the transcriptional differences between the MI and MACS groups

**Figure 3:**
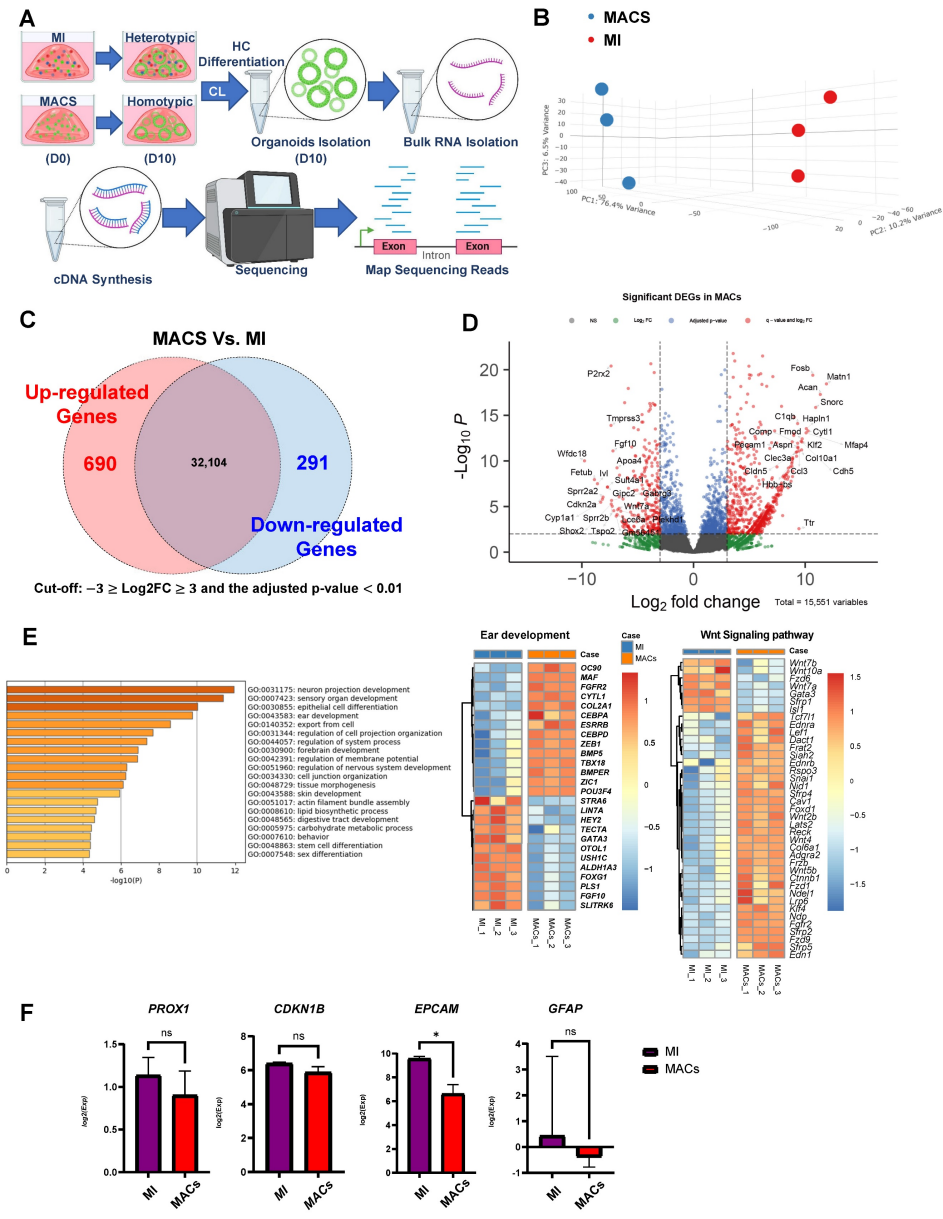
** Transcriptome profiling for MACS isolation. (A)** RNA sequencing (RNA)-seq time points. **(B)** We created a PCA plot to illustrate the similarities and differences in gene expression between the groups of samples created using MACS and MI. Each group is represented by dots with distinct colors, allowing for easy visual differentiation. The plot was constructed using three principal components, each corresponding to one of the axes, which collectively account for the majority of the variance in the data. **(C)** Summary of DEG analysis and significant transcriptional differences between the MACS and MI groups. The numbers of up- and downregulated genes are shown, and overlapping areas in the Venn diagram represent moderate changes in expression. **(D)** Volcano plot representing significant changes in gene expression with a cut-off point of log_2_FC ≥3 or ≤ -3 and q-value < 0.01. The top 20 up- and downregulated genes in the MACS group are ordered according to name. **(E)** The 28 DEGs related to the ear development process (14 up- and 14 downregulated) and 39 DEGs associated with Wnt signaling (7 up- and 32 downregulation) were identified via functional prediction analysis with Gene Ontology and visualized via heatmap clustering. The legend and color key on the right side show the experimental cases and number of expression values, respectively. **(F)** This figure shows the expression levels of key supporting cell markers for hair cell regenerative potentials, and the asterisk indicates significances. *p < 0.05. DEG; Differentially expressed genes, MACS; Magnetic-activated cell sorting, MET; Mechanotransduction, MI; manual isolation, PCA; Principal component analysis, RNA; Ribonucleic acid, RNA seq: RNA sequencing.

We next examined the transcription induced by the homotypic MACS and heterotypic MI cultures using bulk RNA-seq **(Supplementary Figure S7A-D)**. Our analysis revealed distinct transcriptomic differences between the MACS and MI groups **(Figure 3A)**. Specifically, the comparison highlighted a total of 690 upregulated and 291 downregulated differentially expressed genes (DEGs) **(Figure 3B-D)**. Interestingly, there was a notable deficiency (in the MACS group) in the expression of fibroblast growth factor 10 (*Fgf10*), GATA binding protein 3 (*Gata3*), transmembrane protease, serine 3 (*Tmprss3*), and short stature homeobox 2 (*Shox2*). These are vital for cell differentiation and organogenesis **(Figure 3D)** 13, 29-33. Additionally, functional predictions of the downregulated DEGs in MACS indicated that the expression levels of genes related to various aspects of tissue and cell development, including ear development (Gene ontology (GO): 0043583) and tissue morphogenesis (GO: 0048728) **(Figure 3D)**. In terms of transcriptional alterations related to ear development, balanced up and down regulation was observed.

In homotypic MACS, up regulation of fibroblast growth factor receptor 2 (*FGFR2*) and POU class 3 homeobox 4 (*POU3F4*), were observed 34, 35. In terms of transcriptional alterations related to Wnt signaling, mostly up regulation was observed in homotypic MACS **(Figure 3E)**. The expression of key supporting cell markers (EpCAM, PROX1, CDKN1B, and GFAP) involved in the differentiation of supporting cells into auditory hair cells was analyzed, revealing lower EpCAM expression in MACs **(Figure 3F)**. All DEGs in this comparison set are listed, along with the functional classification results respectively **(Supplementary Tables S1 and S2)**.

### Improving MACS-derived LPCs

**Figure 4:**
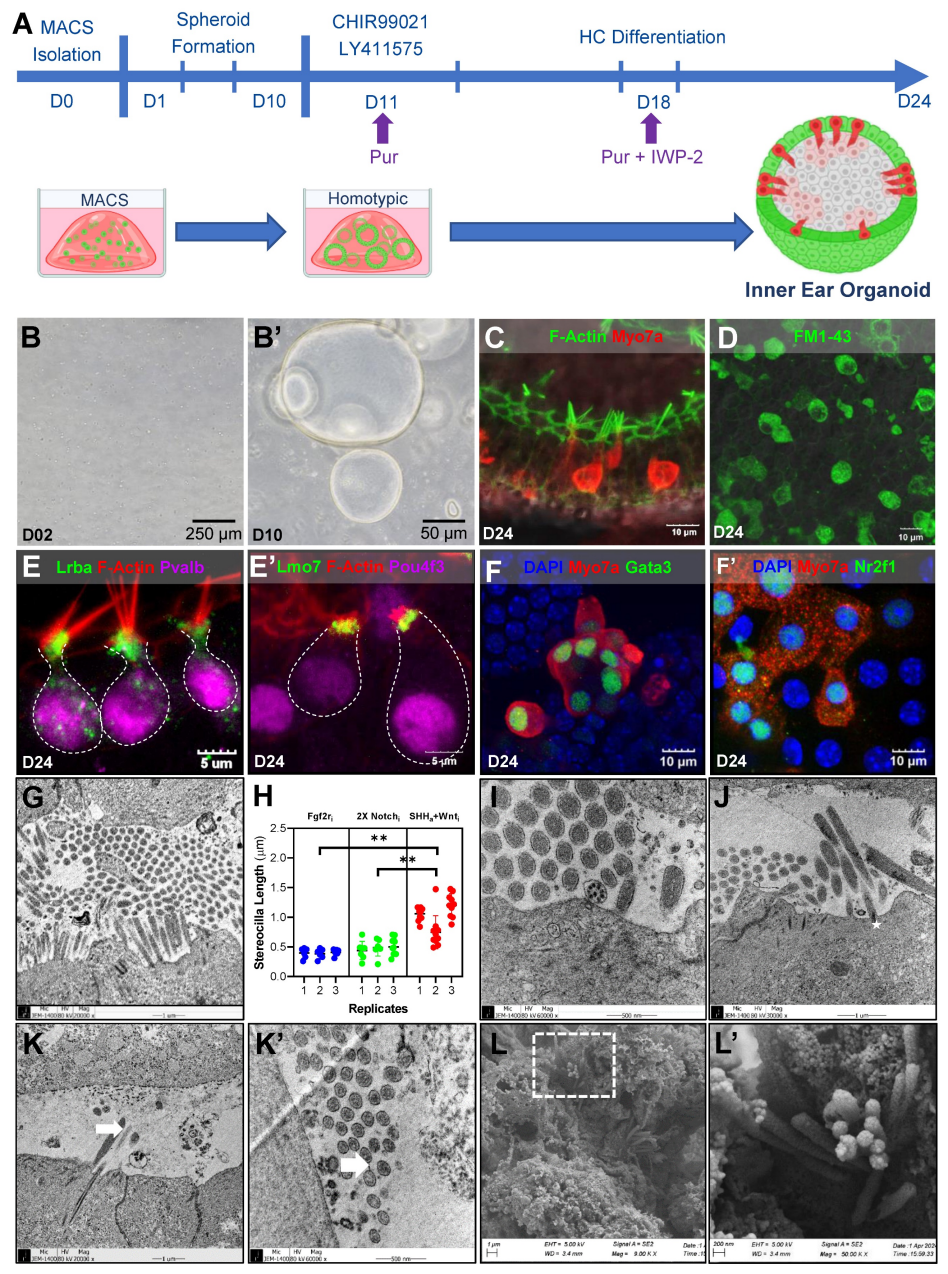
** Schematic and characterization of homogenic cochlear progenitor Lgr5-positive inner ear organoids. (A)** Experimental timeline illustrating the generation of homogenic otic organoids from MACS-isolated Lgr5-positive progenitor cells, including key differentiation stages and treatments (CHIR99021, LY411575, and IWP-2). **(B-B')** Phase-contrast images showing early spheroid formation at D2 and matured otic organoids at D10. **(C)** Immunostaining for F-actin (green) and Myo7a (red) at D24, highlighting the presence of differentiated HCs with organized stereocilia-like structures. **(D)** FM1-43 uptake assay demonstrating functional mechanotransduction in organoid-derived HCs. **(E-E')** Immunostaining of Lrba, Lmo7, F-actin (red), Pvalb and Pou4f3 (magenta) confirming expression of key stereocilia-associated and HC markers at D24. **(F-F')** Immunostaining for DAPI (blue), Myo7a (red), Gata3 (green), and Nr2f1 (green) illustrating the presence of cochlear lineage markers in differentiated organoids at D24. **(G-H)** TEM images of HC-like structures at D24, showing bundled stereocilia arrangements based on cuticular plate with a distinct staircase formation. Stereocilia length measurements in **(H)** reveal increased elongation compared to modified treatment groups, supporting enhanced stereociliary maturation. **(I)** TEM image showing the presence of a possible kinocilium with a distinct 8+1 microtubule configuration, a characteristic feature of developing cochlear HCs. **(J)** TEM image indicating the presence of a rootlet structure (star) at the base of the stereocilia, suggesting anchorage specialization for mature HC function. **(K-K')** TEM images showing possible tip links, viewed from both side and top perspectives (indicated by arrows), suggesting early-stage mechanoelectrical transduction machinery development. **(L-L')** SEM images depicting the ultrastructure of stereocilia bundles in organoid-derived HCs at D24. Higher magnification of the boxed section revealed a distinct "staircase-like" profile and V-shaped arrangement that is typical of inner HCs **(G')**. Scale bar values are indicated in each image. **p < 0.01. D; Day, HCs; Hair cells, Lgr5; Leucine-rich repeat-containing G-protein-coupled receptor 5, MACS; Magnetic-activated cell sorting, MET; Mechanotransduction, MI; manual isolation, Myo7a; Myosin 7a, Pvalb; Parvalbumin, SEM; Scanning electron microscopy, TEM; Transmission electron microscopy.

The results of bulk RNA-seq and evaluation of cellular morphologies clearly indicated that the presence of other cells in the heterotypic culture in the MI group significantly influenced the characteristics and functionality of the differentiated HCs. Therefore, to improve the generation of HCs in the MACS-derived LPCs, we modified the culture conditions **(Figure 4A)**. The modification trials including other groups with different factors such as FGFr inhibitor and Notch inhibitor (G1 and G2 groups) are in the supplementary figures 8 to 10 (see the discussion for further details).

The downregulation of morphogenesis-related genes and Fgf10 **(Figure 3E)**, which is closely related to Shh in the differentiation of the inner ear and other organs 36-38 (as evidenced by RNA-seq) supports the potential role of Shh in LPC differentiation. Given the enrichment of Wnt signaling in MACS compared to MI **(Figure 3E)**, we applied Wnt inhibition in combination with Shh treatment **(Figure 4A)**. Starting at D11, we applied the Shh agonist purmorphamine, together with the Wnt agonist CHIR99021 and the Notch inhibitor LY411575. We then switched the Wnt agonist to a Wnt inhibitor (IWP-2) starting on D18, and continued this treatment until the end of the culture period. Early spheroid formation was evident by Day 2 (D2), with compact cellular aggregates forming in culture **(Figure 4B)**. By Day 10 (D10), the spheroids had expanded, displaying defined lumen-like structures indicative of further epithelial organization **(Figure 4B')**. By D24, the organoids exhibited distinctive hair cell-like morphology, including elongated stereocilia bundles, suggesting differentiation into sensory hair cell-like populations. We performed immunostaining for key hair cell markers at D24. F-actin (green) and Myo7a (red) staining revealed the formation of bundled stereocilia-like structures **(Figure 4C)**. Functional FM1-43 uptake assays demonstrated the presence of mechanotransduction activity, indicating that these cells developed some functional properties associated with mature sensory hair cells **(Figure 4D)**.

Further characterization of hair cell-like populations in the organoids showed robust expression of stereocilia-associated proteins, including Lrba and Lmo7 **(Figure 4E, E')** in the cuticular plate. Additionally, expression of Parvalbumin (Pvalb), a calcium-binding protein **(Figure 4E, S10)**, and Pou4f3 **(Figure 4E')**, a transcription factor essential for hair cell differentiation, were co-localized with these stereocilia markers, supporting the notion that the cells were committed to a hair cell fate. To further verify the cochlear identity of these cells, we examined the expression of Gata3 and Nr2f1, two transcription factors implicated in cochlear sensory cell fate specification. These markers were strongly expressed in Myo7a-positive cells, confirming the commitment of the organoid-derived cells to a cochlear hair cell lineage **(Figure 4F, F')**.

We also investigated the ultrastructural morphology of the HCs in the organoids at D24 using electron microscopy **(Figure 4G-L)**. Shh-treated organoids developed into HCs with well-formed stereociliary bundles **(Figure 4G)**, as also revealed by F-actin staining with a mean length of 1.0 ± 0.28 µm **(Figure 4H)**, and were longer than those of the other modified-treatment groups. Among the clusters of stereocilia, we observed structures that may have been microtubules, indicating the potential presence of a kinocilium **(Figure 4I)**. Typically, kinocilia are characteristic of vestibular HCs with a 9 + 2 microtubule configuration. However, a magnified TEM image of the potential kinocilium in our organoid showed a 1 + 8 microtubule configuration, indicating the presence of HCs resulting from the transdifferentiation of supporting cells 39.

Our observations of such features were supported by the expression levels of kinociliary markers in certain organoids **(Figure S11)**. Additional stereocilia structures were observed, including rootlets **(star, Figure 4J)** that connected the stereocilia to the cuticular plate (which was not observed in MACS control; **Supplementary Figure S12**), further confirming the presence of differentiated HCs in the organoids. Possible ciliary links (white arrow) were seen connecting the stereocilia to one another, as seen from the side- **(Figure 4K)** and top-view **(Figure 4K')** TEM images. We further examined the surface morphology of the HCs in the organoids using scanning electron microscopy (SEM) to obtain additional insight regarding their organization. This revealed the intricate organization and structure of the HCs in the organoids, with well-formed stereocilia bundles (Figure 4L) with a distinct "staircase-like" profile, as well as a V-shaped arrangement of HCs **(Figure 4L')**. These findings further indicate that the modified culture conditions led to the differentiation of organoids with inner ear HCs that had comparable morphology to native HCs.

### The transcriptional differences between the MACS control (D24) and Shh-treated groups

**Figure 5:**
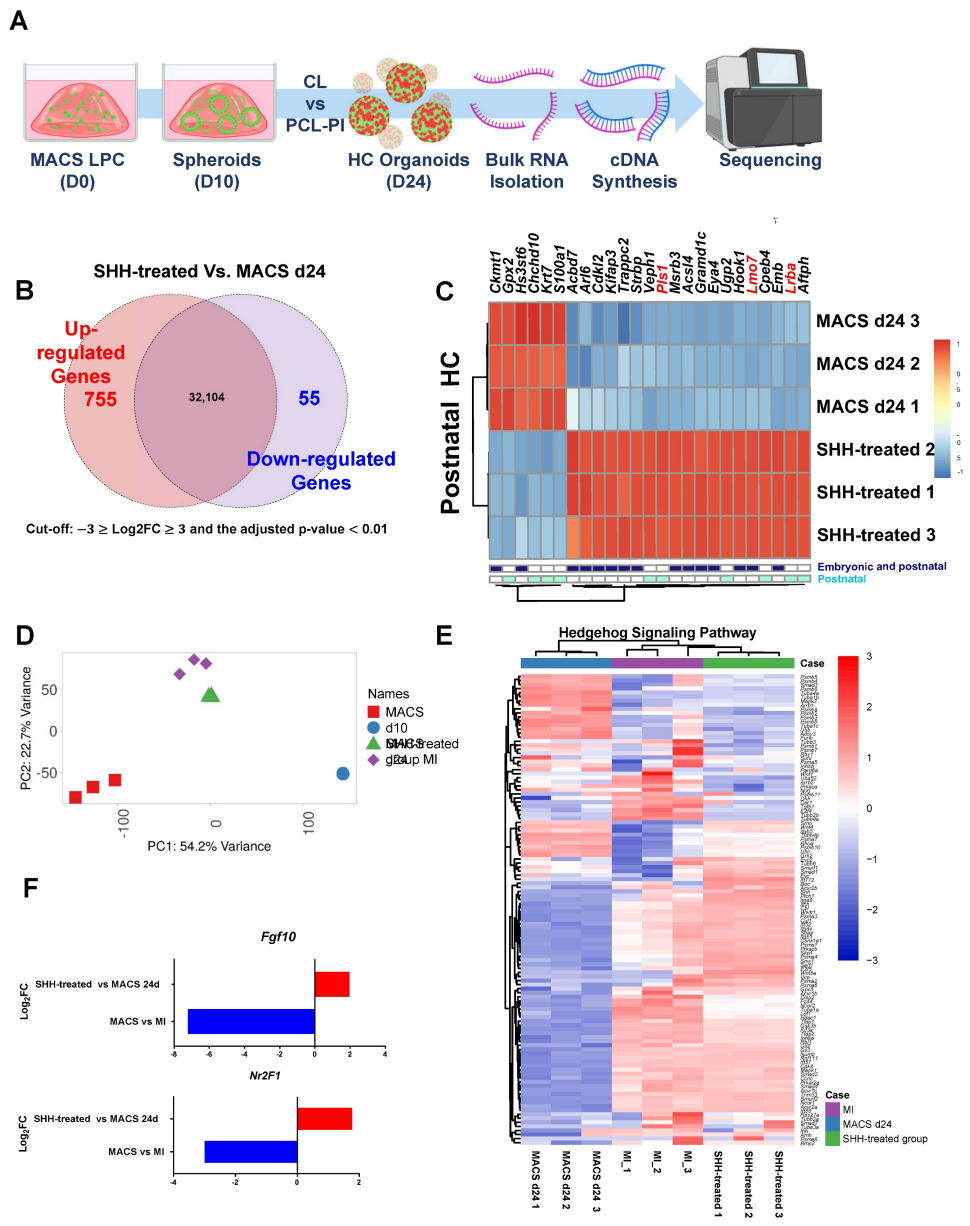
** Integrative analysis of transcriptional transition for inner HC development. (A)** RNA-seq time point. **(B)** This analysis highlights the transcriptional differences between the MACS group and the Shh agonist group and enumerates the up- and downregulated genes in each group. A Venn diagram shows overlapping genes with moderate changes in expression. **(C)** Differences in gene expression between Shh-treated groups and MACS control were validated for genes specifically expressed in embryonic and postnatal auditory cells, as highlighted in previous studies. Most of the genes involving inner ear HC and stereocilia formation were highly expressed via molecular biological modification, indicating that they are involved in auditory cell development. Bold red regions indicate genes that were significantly differentially expressed in stereocilia. **(D)** The PCA plot illustrates the transcriptomic positioning and homology of each sample based on the global pattern of gene expression. Each sample is distinguished by a unique color and symbol on the right side of the plot, four-group comparison (MI, MACS D10, Shh-treated group, and MACS D24). **(E)** Shh agonist-induced transcriptional transitions of genes related to inner HC development are shown. The dendrogram on the top shows the hierarchical similarity between samples and indicates that Shh-treated group had similar gene expression to the MI group. The legend and color key on the right side of the plot show the expression values of the experimental groups. **(F)** Comparison of significant changes in *Fgf10* and *Nr2f1* expression associated with cochlear development. The height of the bar plot on the x-axis (log_2_FC) represents the changes in expression for the groups. MACS; Magnetic-activated cell sorting, MET; Mechanotransduction, MI; manual isolation, PCA; Principal component analysis, RNA; Ribonucleic acid, RNA-seq: RNA sequencing, Shh; Sonic Hedgehog.

The bulk RNA-seq data revealed clear molecular biological differences between the MACS control and Shh-treated groups at D24 **(Supplementary Figure S13A, B)**. A total of 755 upregulated and 55 downregulated DEGs were identified in the comparison **(Figure 5A, B).** Notably, genes specifically enriched in sensory organ development (GO:0007423) were also identified **(Supplementary Figure S13C)**.

Among the 533 known HC-related genes 40, dominant gene up-regulations in Shh-treated group were observed **(Supplementary Figure S13D, Table S4)**. Among them, 19 HC-related genes were significantly up-regulated in the Shh-treated group, including *Eya4* (EYA transcriptional coactivator and phosphatase 4), *Lrba* (lipopolysaccharide-responsive beige-like anchor protein), *Lmo7* (LIM-only protein 7), and *Pls* (plastin 1), which are essential for sensory HC and stereocilia and cuticular plate development 41-45. In contrast, six genes, including *Gpx2* (glutathione peroxidase 2) and *S100a1* (S100 calcium-binding protein A1), were highly expressed in the MACS control group. Their roles in cochlear HC differentiation remain unclear **(Figure 5C)**.

In the four-group comparison (MId10, MACSd10, Shh-treated group, and MACSd24), the Shh-treated and MI groups exhibited similar gene expression patterns, suggesting a shared regulatory mechanism **(Figure 5D)**. Similarly, when comparing Shh-specific gene expression across groups, the Shh-treated group showed a gene expression pattern similar to that of the MI group **(Figure 5E, Supplementary Table S5)**. Additionally, among the downregulated genes in the prior bulk RNA-seq analysis of MI vs. MACS at D10, *Fgf10* (an Shh-responsive factor) and *Nr2f1* (previously shown to be upregulated in cochlear organoids 46) exhibited significantly increased expression in the Shh-treated group compared to the MACS control **(Figure 5F)**.

### Single-cell RNA-seq statistics and cell characterization

Using scRNA-seq, we quantified and analyzed cell type-specific transcript expressions in organoids. A total of 10,997 cells were obtained from the Shh agonist group, and scRNA-seq analysis yielded an average of 28,597 reads per cell. A total of 22,833 genes were detected, with an average of 7,009 unique molecular identifiers (UMIs) per cell when compared to the mouse reference genome (GRCm39/mm39; Jun, 2020), achieving 96.7% coverage. Five major clusters with comparable expression to the mean gene expression levels were identified. A detailed statistical summary is given in **Supplementary Table S6**, and **Figure S14A**. Transcriptomic profiling of the mouse cochlea was based on the expression levels of standard markers and the cell types, then subdivided into five significant ensembles based on the origin of the cochlear subregion 47, 48. In detail, we used the scRNAseq data to identify a cell ensemble of the neural sensory epithelium with supporting cells (SCs) (expressing *Otogl*, *Gas2*, *Gkb2*, and *Gjb6*), HCs (expressing *Capb2*, *Ush2a*, *Ush1g*, and *Pou4f3*), and glial cells (GCs) (expressing *Pou3f4*, *Slc9a3r2*, *Maft*, and *Bhlhe22*). The remaining cochlear cell types formed a cell ensemble that we termed "surrounding structures (SSs)", the cell type of which was revealed by analysis of seven genes, including *Pou3f4*, which is predominantly expressed in the ear mesenchyme that surrounds the ear vesicles.

**Figure 6:**
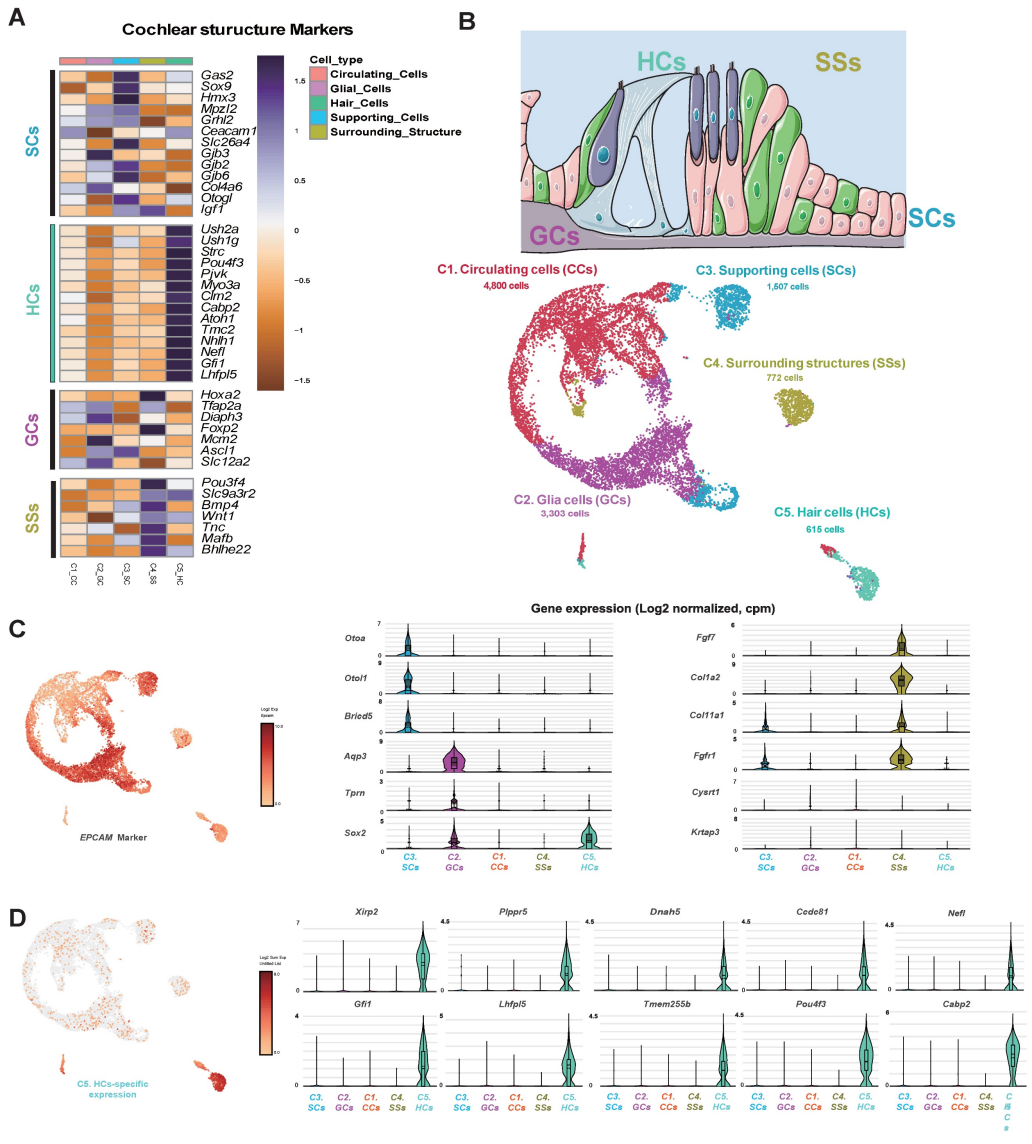
** Cell-type clustering of differentiated HCs exposed to an Shh agonist as revealed by scRNA-seq. (A)** Heatmap of the cochlear structural markers. Increased color intensities of the scale bars indicate higher gene expression levels in the various plots, as revealed by the log_2_ FC values. **(B)**. A sketch of the cochlea showing the principal cell-type ensembles on which we focused. The UMAP plots for five types of cells isolated from differentiated HCs after exposure to an Shh agonist. **(C)** The UMAP plot for the *Epcam* marker, which plays a vital role in the 'Organ of Corti' that maintains structural organization and differentiation. Light brown: Cells expressing the genes. The violin plots show the expression levels of the gene subsets that classify the cell types. **(D)** A UMAP plot of the top 10 most highly expressed genes in the C5 HC cluster. Violin plots of HC cluster-specific features based on the differential expressions of the top 10 genes. HCs; Hair cells, Shh; Sonic Hedgehog; UMAP; Uniform manifold approximation and projection.

Finally, the remaining cochlear cell types subjected to single-cell analysis were characterized by the expression of immune system-related genes and structural proteins of the connective tissue extracellular matrix, including collagen and keratin **(Figure 6A)**. Dimension reduction for clustering was carried out using the t-distributed stochastic neighbor embedding (t-SNE) and Uniform Manifold Approximation and Projection (UMAP) algorithms, and the results from the UMAP algorithm were selected for further analysis. Of the 10,997 cells, 3,303, 1,507, 772, and 615 were GCs, SCs, SSs, and HCs, respectively **(Figure 6B)**. Comparisons with previously published data, including those on 334 genes that are preferentially expressed in postnatal HCs 36, revealed that 48 genes exhibited significantly higher expression levels in HC clusters. The results suggest that cells in HCs may play important roles in the development, maintenance, and/or function of auditory cells **(Figure S14B)**. Examination of differentially expressed genes among the clusters identified 521 up-regulated and 661 down-regulated gene sets. Typically, HCs contained 233 and 443 up- and down-regulated genes, respectively (cut-off: log2FC ≥ 2, ≤ -2, p < 0.01). A detailed list of the genes and functional profiling are given in Supplementary Table S7 and S8.

### Evaluation of scRNA-seq information

Epithelial cell adhesion molecule (*EPCAM*) 49, an essential epithelial marker, was upregulated in all clusters of the Shh-treated group, confirming their derivation from inner ear epithelial cells **(Figure 6C)**. We first identified genes specifically expressed in SCs and found that *Otoa*, a tectorial membrane protein located around HCs; *Otol1*, which encodes the Otolin protein; and *Brcid5*, which encodes a protein involved in cell differentiation via the BRICHOS domain, were specifically expressed. In GCs, *Aqp3*, which encodes Aquaporin, a cell membrane water channel protein, and *Tprn*, which encodes Taperin, a protein supporting cell structure maintenance, were expressed at significant levels. Additionally, *Sox2*, a transcription factor crucial for maintaining stem cell pluripotency and necessary for nervous system development, was also expressed. However, its expression was higher in HCs than in non-sensory cells. The Cluster 5 HCs exhibited a gene expression profile that differed from those of other clusters, including upregulation of the previously identified HC markers *Xirp2*, *Nefl*, *Lhfpl5*, *Pou4f3* 47, *Dnah5* 40, *Gfi1* and *Cabp2* 50; and also the newly identified genes *Plppr5* 51, *Ccdc81* 52, and *Tmem255b* 53 that are involved in phospholipid metabolism, cytoskeletal interactions, and cellular signaling **(Figure 6D)**.

The gene expression profile of HC clusters was characterized by the enrichment of cochlea-specific markers, including *Fgf8, Tbx2, Brip1, Lrrn1,* and *Zfp324*, which distinguish HCs from other cell types. However, the expression levels of the vestibular HC markers *Oosp2* and *Tex14* suggest a degree of heterogeneity within the HC population. The upregulation of inner ear-related genes 54 across all clusters is evident in **Figure S14C**. A comparison of gene expression profiles between inner and outer HCs yielded inconclusive results **(Supplementary Figure S14D)**. GO analysis confirmed that HC differentiation in Shh-treated organoids was characterized by subcell-specific gene expression rather than uniform HC marker expression. The mechanoreceptor differentiation (GO:0042490) and cilium organization (GO:0044782) pathways were significantly enriched in the HC cluster. Furthermore, certain stereocilia-related genes (GO:0032420) and non-motile cilium components (GO:0097730) were highly expressed, highlighting the development of key sensory structures **(Supplementary Figure S14E, Supplementary Table 10)**.

**Figure 7:**
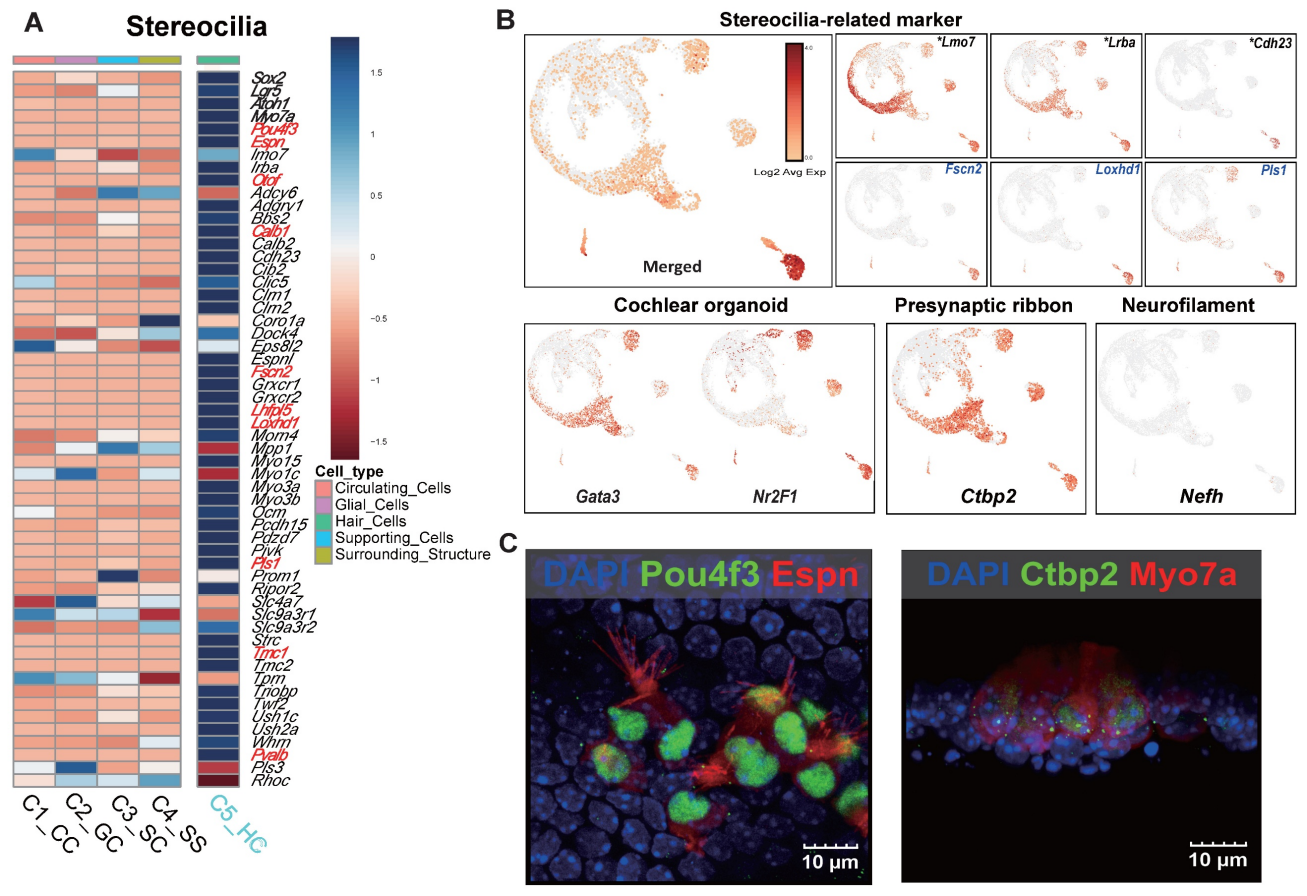
** The single-cell gene expression features of stereocilia and the electrophysiological results obtained using the microelectrode assay.** (A) A heat map of stereocilia-related gene expression. The genes in red exhibited log_2_FC values > 5. (B) The UMAP plot of stereocilia-related markers. The asterisks and blue gene names indicate well-known and novel genes identified in the scRNA-seq study, respectively. Representative cellular markers of the cochlear organoid, presynaptic ribbon, and neurofilament are shown. (C) Cells in Shh-activated and Wnt-inhibited IEO stained with Ctbp2 and Espin at the end of the 2-week differentiation period (D24). D; Day, scRNA-seq.; Single cell RNA sequencing, Shh; Sonic Hedgehog, UMAP; Uniform manifold approximation and projection.

Functional classification identified an HC cluster with a developmental trajectory similar to that of mature HCs. This cluster exhibited strong expression of specific stereocilia-related genes (*Espn, Myo7a, Pou4f3, Otof,* and *Smpx*), suggesting that it represents a distinct stereocilia-forming population. Additionally, newly identified gene candidates associated with stereociliary stability and mechanotransduction (*Fscn2, Loxhd1*, and *Pls1*) and genes that were related to the cuticular plate which were upregulated in the prior bulk RNA-seq comparison (*Lrba*, and *Lmo7*) were significantly expressed, despite being previously underrepresented in scRNA-seq analyses **(Figure 7A, B)**. Genes previously reported as highly upregulated in cochlear organoids 46 were overexpressed across multiple HC clusters **(Figure 7B)**. Notably, *Nr2f1*, which was downregulated in the MACS group but showed increased expression following Shh application, suggests a transcriptional response induced by morphogen stimulation **(Figure 5F, Figure 7B)**. Additionally, genes associated with synaptic function and spiral ganglion connectivity were upregulated, particularly *Ctbp2*, a key marker of the presynaptic ribbon. This gene was strongly expressed across multiple clusters, including the HC cluster. Epifluorescence analysis confirmed that the morphologies of the presynaptic ribbon and stereocilia were distinct, further supporting functional maturation **(Figure 7B, C)**. Interestingly, neurofilament H (*NefH*), a crucial structural component of axonal nerve fibers that transmit signals between sensory neurons and IHCs, was absent in Shh-induced organoids **(Figure 7B)**.

### Electrophysiological study

**Figure 8:**
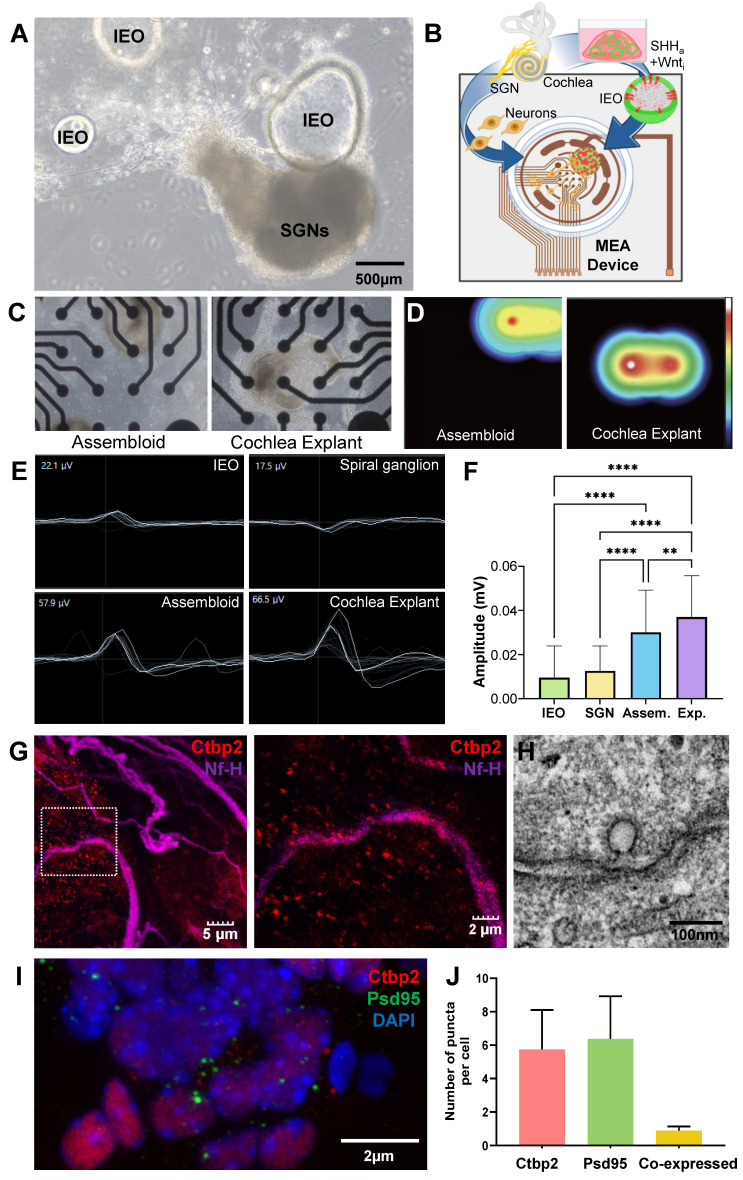
** Functional evaluation of assembloids formed by Shh-treated MACS- IEOs and SGN explants. (A)** Brightfield image of an assembloid formed by co-culturing Shh-treated MACS-derived IEOs with SGN explants, showing direct cellular interaction. **(B)** Schematic illustration of the experimental setup, highlighting the integration of IEO, SGNs, and a MEA device for functional assessment. **(C)** Representative brightfield images of the assembloid (left) and SGN explant (right) cultured on the MEA plate for 10 days. **(D)** Heatmap representation of MEA signals, demonstrating higher electrophysiological activity in assembloids, similar to cochlear explants. **(E-F)** MEA recordings from different culture conditions, including organoid-only, SGN-only, assembloids, and cochlear explants. The assembloid group exhibited higher spontaneous activity (57.9 µV) compared to organoid-only (22.1 µV) and SGN-only cultures (17.5 µV), approaching levels observed in cochlear explants (66.5 µV). **(G)** Immunostaining of assembloids, showing synaptic connectivity between CtBP2-positive (red) presynaptic ribbons, and NF-H-labeled (purple) neuronal projections. **(H)** TEM images indicate synaptic ribbon like structure. **(I and J)** Confirmation and quantification of synaptic markers (CtBP2 and PSD95) showing co-localization in assembloids, supporting enhanced synaptogenesis. D; Day, IOEs; inner ear organoids, MACS; Magnetic-activated cell sorting, MEA; Microelectrode array, SGN; Spiral ganglion neuron, Shh; Sonic Hedgehog, TEM; Transmission electron microscopy.

To evaluate the functional integration of Shh-treated MACS-derived IEOs, assembloids were generated by co-culturing IEOs with primary spiral ganglion neurons (SGNs) under optimized conditions. Brightfield imaging revealed direct physical interactions between the IEOs and SGNs after 10 days of co-culture, suggesting potential synaptic integration **(Figure 8A)**. To assess the electrophysiological activity of these assembloids, we utilized microelectrode array (MEA) recordings **(Figure 8B)** and compared the results to baseline controls (organoid-only, SGN-only cultures, and cochlear explant). Heatmap representations confirmed that assembloids displayed strong localized activity patterns comparable to cochlear explants, reinforcing their functional maturation **(Figure 8C, D and videoclips 1 and 2)**. The MEA recordings indicated that assembloids exhibited significantly higher spontaneous electrical activity (57.9 µV) compared to organoid-only (22.1 µV) and SGN-only (17.5 µV) cultures. The recorded activity in assembloids closely approached the values observed in cochlear explants (66.5 µV), suggesting functional integration between IEO-derived hair cells and SGNs **(Figure 8E, F)**.

To confirm synaptic connectivity, immunofluorescence staining was performed for CtBP2 (red, presynaptic ribbon protein), and NF-H (purple, neuronal projections) **(Figure 8G)**. Merged images demonstrated the presence of synaptic contact points between hair cell-like structures and SGNs, supporting the hypothesis of functional neural integration within assembloids. TEM image further revealed presynaptic ribbon like structure adjacent to the cell membrane **(Figure 8H)**. Additionally, confirmation and quantification of pre (CtBP2) and post synaptic markers (PSD95), including co-localization, supports the synaptic connection in assembloids **(Figure 8F, J)**. These findings suggest that the co-culture of Shh-treated IEOs and SGNs enhances synaptogenesis, leading to functionally active sensory-neuronal assembloids that exhibit electrophysiological properties comparable to native cochlear tissue.

## Discussion

In this study, we investigated the molecular and functional mechanisms underlying stereocilia maturation in homogenic Lgr5-positive cochlear progenitor-derived IEO. Using transcriptomic and functional analyses, we demonstrated that Shh signaling, in combination with Wnt inhibition, plays a crucial role in promoting the formation and functional maturation of stereocilia in these organoids. These findings provide important insights into cochlear HC development and highlight a potential strategy for advancing inner ear regenerative therapies.

LPCs were first used to generate inner ear organoids a decade ago 21, 55, and this technology has been gradually improved over time to enable the development of cells that more closely mimic cochlear HCs 18, 19, 23, however, the challenge of achieving fully mature stereocilia remains a critical barrier to functional regeneration. Currently, the optimal method of HC production involves starting with a heterotypic culture of the cochlear sensory epithelia and adding small molecules and growth factors to induce differentiation 55. One of the most significant outcomes of this study was the identification of morphological and key transcriptional differences between heterotypic and homogenic Lgr5-derived organoids. Prior publications using isolated progenitor cells did not report distinct stereocilia features, rather only short and less distinct/merged Espin or F-actin expression) 54-56 , which are crucial to mechanoelectrical transduction in HCs. Our electron microscopic analysis of organoids from homotypic culture indicated no distinct features and only possible microvilli **(Figure S12)**. However, by modulating developmental signaling pathways, particularly through Shh activation and Wnt inhibition, we observed improved stereocilia morphology, as confirmed by ultrastructural imaging as well as significantly heightened expression of stereocilia and especially cuticular plate-related genes (*Pls*, *LMO7*, *LRBA*). This information led the authors to predict that Shh treatment resulted in mature stereocilia by improving cuticular plate integrity, as confirmed by epifluorescence analysis, bulk RNA sequencing, single-cell RNA sequencing, and TEM (see the graphical abstract for illustration) 26, 57.

Whether the differences between MI- and MACS-derived organoids are related to the lack of other supporting cells, degenerating HCs, or neurons is unclear. The presence of non-Lgr5-positive supporting cells in the cochlear sensory epithelia within heterotypic cultures introduces additional cellular interactions that can obscure the specific mechanisms driving LPC differentiation into hair cells. Supporting cell populations such as p27^Kip1-positive 58, Sox2-positive 59, Prox1-positive 60, Hensen's 61, and EpCAM-positive 62 cells, have been shown to exhibit varying degrees of plasticity and regenerative potential, contributing to hair cell formation under specific conditions. In a heterotypic environment, these non-LPCs may influence the differentiation trajectory of LPCs through paracrine signaling, indirect transcriptional regulation, or cellular competition, making it challenging to isolate the intrinsic differentiation potential of LPCs. Indeed, bulk RNA-seq analysis at D10 revealed that EpCAM expression was significantly higher in the MI group (Figure 3F), suggesting a possible role for EpCAM in the differences observed in the final differentiation outcomes between the MI and MACS groups. Additionally, supporting cells with latent regenerative capacity could give rise to hair cells independently, confounding our ability to determine whether LPC-derived organoids are uniquely responsible for hair cell generation. By employing a homotypic culture system, where only LPCs are present, we can eliminate these confounding factors and precisely investigate the intrinsic differentiation capacity of LPCs. This approach provides a controlled and reproducible environment for studying the molecular pathways governing LPC differentiation, ensuring that observed hair cell formation is directly attributable to LPCs rather than external influences from non-LPC populations. Furthermore, our data permit us to speculate that the stimuli from other cells are mostly related to morphogenesis, which is in closely related to the formation of stereocilia.

Beyond Shh signaling and Wnt inhibition, we explored additional strategies to improve HC differentiation as part of our supplementary investigations. We tested various culture conditions in different experimental groups to optimize HC production. In the first group, we inhibited Fgf2 at the early stage of spheroid formation to mimic the downregulation of Fgf2 observed in our bulk RNA-seq analysis. Using the Fgf2 receptor inhibitor AZD4547, we aimed to determine its effect on differentiation. A modified condition was also established, where AZD4547 was applied at D11 instead of D1 **(Figure S8A)**. In another experimental approach, we tested whether further increasing Notch inhibition would enhance HC differentiation, as our transcriptomic data suggested a role for Notch signaling in limiting HC formation 46, 63-65. We doubled the concentration of the γ-secretase inhibitor LY411575 at D11 and created an additional group where LY411575 was also increased at D7 (Figure S8A). However, our analyses showed no significant differences in organoid size, Myo7a-positive cell morphologies, or F-actin distribution between different time points of AZD4547 and LY411575 application **(Figures S9)**. Further characterization revealed that Fgf2 inhibition significantly reduced spheroid size, suggesting that Fgf2 is essential for the early formation and growth of HC organoids 36, 37, 66. In contrast, the spheroids treated with increased Notch inhibition and Shh activation were similar in size to control groups **(Figures S8B, C)**. Transmission electron microscopy (TEM) analyses of the Fgf2-inhibited and increased Notch-inhibition groups revealed that only microvilli protruded into the lumen of the organoids, rather than stereocilia-like structures **(Figure 4H and S12)**.

The interplay between Shh signaling and Wnt inhibition in cochlear development has been an area of interest in regenerative medicine. Wnt signaling is known to regulate progenitor proliferation and HC specification, while Shh influences spatial patterning and differentiation 57, 65. Our results suggest that precise modulation of these pathways is critical for enhancing stereocilia maturation and achieving functional HC regeneration. The ability to refine these conditions in vitro could improve differentiation protocols for future applications in inner ear regeneration.

The results of our scRNA-seq analysis provided deeper insights into the cell populations affected by Shh activation and Wnt inhibition. Compared to untreated organoids, those exposed to Shh and Wnt inhibition exhibited strong upregulation of *EPCAM* in all clusters suggests that Shh-treated cells originated from inner ear epithelial cells, which serve as the foundational cell type during HC and supporting cell differentiation. Cluster 5 HCs exhibited a gene expression profile that differed from those of other clusters, including upregulation of the newly identified genes *Plppr5***,** *Ccdc81***,** and *Tmem255b* involved in phospholipid metabolism, cytoskeletal organization, and cellular signaling. Additionally, enrichment of the vestibular HC markers *Oosp2* and *Tex14* suggests that this subpopulation of HCs exhibits unique molecular characteristics, highlighting a previously unrecognized diversity in terms of HC differentiation within cochlear organoids. Also, the HC cluster displayed robust expression of certain stereocilia-related genes (*Espn*, *Myo7a*, and *Smpx*), reinforcing the roles played in stereociliary formation 47 The newly identified candidate genes *Fscn2*, *Loxhd1*, and *Pls1* that contribute to structural stereociliary stability and mechanotransduction, were significantly enriched in the HC cluster, further distinguishing the molecular profile thereof.

Another important factor, *Fgf10,* was found to be required for inner ear formation and that its interaction with Shh has been implicated in morphogenesis 67. For example, hedgehog and FGF signaling provide the complementary cross-inductive signals required for limb regeneration. Specifically, posteriorly localized Shh is required for the maintenance of anteriorly localized FGF8 expression and the non-localized expression of FGF9, FGF10, and FGF17 68 . These interactions are mostly observed during the morphogenesis of respiratory epithelium 38, 69 , which shares some morphologic features with sensory HCs, including those related to the ciliated epithelium. However, few studies have examined how *Fgf10* acts as a signaling molecule in inner ear cells. Indeed, this study is the first to show that *Fgf10* responds to Shh in a way that influences stereocilia morphology. These findings suggest that Shh facilitates both HC differentiation and structural specialization. The latter is a critical aspect of functional maturation.

Turning to functional maturation and neural connectivity, enrichment of the *Ctbp2* presynaptic ribbon marker in multiple HC clusters indicates that these cells may engage in synaptic specialization. Epifluorescence analysis further confirmed the distinct morphologies of the presynaptic ribbon and stereocilia, supporting the hypothesis that Shh-treated organoids exhibit early signs of functional maturation. This prompted us to perform electrophysiological assessments using assembloids of Shh-treated IEO with spiral ganglion primary cultures, to determine whether the IEO supported auditory signal transmission. Microelectrode array recordings confirmed the presence of neural activity and mechanoelectrical transduction, supporting the notion that these cells had developed key functional properties required for sound perception. The enhancement of stereociliary structure and function further supports the potential of these organoids as models for studying HC biology and as a platform for therapeutic development.

It is still unclear whether the HCs with good stereocilia morphology observed in the current study are closer to cochlear HCs or vestibular HCs. Even though these cells are from the cochlea, a previous study involving scRNA-seq of MI-obtained cells indicated that vestibular HC features could be observed in MI-derived tissue. Upon comparison of the gene expression of Shh-treated group organoids in scRNA-seq data from an MI group in a previous report 70, minimal expression of cochlear/vestibular HC markers was observed, indicating that the HC-like cells were premature. Considering the stereocilia morphology visualized by SEM, *Gata3* expression in *Espin*-positive cells and downregulation of *Gpx2* in the Shh group (as revealed by bulk RNA-seq) suggest a cochlear lineage. Further maturation of these organoids will require the establishment of a maturation protocol. Kinocilium is present in both transdifferentiated and developmental HCs, and constitutes evidence of both prematurity and continued stereociliary maturation 39. True kinocilium is never observed in MACS control cells but Shh induction may possibly facilitate kinocilium expression and further stereociliary development.

While our findings represent a significant step forward, several challenges remain. One limitation is the need for extended maturation time to fully replicate the architecture of native cochlear hair cells. Future studies should explore long-term culture conditions and additional signaling modifications to further enhance HC maturation. Moreover, integrating these findings with in vivo models will be essential for validating their translational potential.

## Conclusion

The research reveals that Shh signaling alongside Wnt inhibition significantly improves stereociliary maturation within IEO derived from homogenic cochlear LPCs. Integrated transcriptomic and functional analyses helped us uncover principal molecular pathways that control the formation of mechanosensitive hair cell-like structures. The study shows that the cuticular plate is crucial for stereocilia organization and stability because it triggers a significant increase in essential structural proteins. The modulation through Shh treatment led to stereocilia that had clearer structural definitions and improved cuticular plate integrity confirmed by ultrastructural imaging techniques combined with single-cell RNA sequencing and electrophysiological evaluations. The IEO demonstrated neural integration potential through synaptic connections when co-cultured with SGNs which enabled functional restoration capabilities. These findings offer valuable insights into the molecular mechanisms underlying inner ear regeneration and provide a robust platform for advancing cell-based therapies for sensorineural hearing loss.

## Methods

### A. Mouse models

Lgr5-EGFP-IRES-CreERT2 (Stock 008875) mice were purchased from The Jackson Laboratory (Bar Harbor, MN, USA). Wild-type C57BL/c6 mice were purchased from Nara Biotech (Seoul, Korea). Both male and female post-natal D1-2 (P1- P2) mice were used in all experiments. Animal care and experiments were performed according to a protocol approved by the Animal Care and Use Committee of Dankook University (approval number DKU-19-026 and DKU-21-014). The cochlear explant method was based on a previously published protocol 71.

### B. DNA isolation and genotyping

While homozygous mice are not viable, heterozygous Lgr5-EGFP-IRES-CreERT2 mice are both viable and fertile. They harbor an Lrg5-EGFP-IRES-creERT2 "knock-in" allele that abolishes Lgr5 (Gpr49) gene function, with expression of the EGFP and CreERT2 fusion protein from the Lgr5 promoter/enhancer elements. To determine mutants against wild-type progenies after breeding, DNA was isolated from pups using the NaOH extraction method (Truett GE et al. 2000, Biotechniques 29(1):52-54) for genotyping.

Briefly, a 2-mm section of the tail of each mouse pup was removed and placed into an Eppendorf tube or 96-well plate, to which 75 µL of 25 mM NaOH/0.2 mM EDTA was added. Then, the samples were placed in a thermocycler at 98ºC for 1 hour, after which the temperature was reduced to 15°C until it was time to proceed to the next step. Next, 75 µL of 40 mM Tris HCl (pH 5.5) was added, and the samples were centrifuged at 4,000 rpm for 3 minutes. The supernatant was collected and aliquoted for PCR (2 µL undiluted, or 2 µL of a 1:100 dilution/reaction).

Qualitative PCR was performed using the following primer sequences recommended by Jackson Laboratories: mutant reverse (5′- CTG AAC TTG TGG CCG TTT AC-3′), wild-type reverse (5′- GTC TGG TCA GAA TGC CCT TG-3′), and common (5′- CTG CTC TCT GCT CCC AGT CT-3′). The primers were obtained from Bioneer (Daejeon, Korea). The PCR products were separated on a 2% agarose gel, stained with EtBR, and photographed. Two PCR products were expected: a 386-bp product from wild-type alleles and a 119-bp product from the mutated alleles.

### C. LPC isolation

LPCs were isolated using either MI or MACS. For MI, cochleae from at least three P1-P2 mouse inner ears were dissected in phenol red-free Hanks' balanced salt solution (HBSS) with calcium and magnesium (Gibco, #14025134). The cartilage was opened, the stria vascularis was removed, then the sensory epithelia was detached from the modiolus and incubated in a 100-200-μl droplet of Matrisperse Cell Recovery Solution (Corning, Corning, NY, USA) for 1 h at room temperature. This non-enzymatic solution disintegrates the extracellular matrix of the sensory epithelia, enabling the subsequent separation of HCs and supporting cells from the mesenchyme and neurons. Separation was performed using a stripping method in which one pair of forceps was used to hold the sensory epithelium in place while another pair was used to gently remove the layer of HCs and supporting cells. The forceps holding the intact sensory epithelium were then used to remove the mesenchyme and neurons from the dish, while the HCs and supporting cells remained in the dish for subsequent collection. The cells were then collected, centrifuged for 5 min at 0.5 × g, and incubated in TrypLE (Gibco) for 20 min at 37°C. After additional centrifugation for 5 min at 0.5 × g, the dissociated cells were suspended in HBSS, triturated 50-80 times using a 200-μl pipette tip, and strained using a 40-micron cell strainer to produce a single-cell suspension.

For MACS isolation, whole cochleae from at least 40 mouse inner ears were dissected in Dulbecco's phosphate-buffered saline (DPBS) and incubated in 2 mg/mL collagenase A (Roche/Merck) for 1 h at 37°C before being centrifuged for 5 min at 300 × g. The dissociated cells were suspended in DPBS, triturated 50-80 times using a 1,000-μl pipette tip, and strained using a 40-micron cell filter to produce a single-cell suspension. The single-cell suspension was successively incubated with rabbit anti-mouse Lgr5 polyclonal antibody (Miltenyi Biotech, Bergisch Gladbach, Germany) diluted 1:100 with PBS for 45 min at room temperature. After being washed with cold MACS buffer (Miltenyi Biotech) and centrifuged, the cell pellet was resuspended in MACS buffer and incubated for 15 min at 4°C with 20 µl of anti-rabbit IgG microbeads (Miltenyi Biotech) per 10^7^ total cells. The Lgr5-positive cells were sorted using an LS magnetic column (Miltenyi Biotech) placed in the magnetic field of a separator according to the manufacturer's instructions.

The isolated LPCs obtained via MI and MACS were assayed against a phycoerythrin (PE)-conjugated Lgr5 antibody and analyzed for purity using a CytoFLEX flow cytometer (Beckman Coulter, Brea, CA, US).

### D. Sphere culture and differentiation assay

LPCs isolated via MI and MACS were resuspended in 100% Matrigel Basement Membrane Matrix (growth factor reduced, LDEV-free; Corning). Five droplets (30 μl) of Matrigel were placed in each well of a 6-well plate. The plate was incubated at 37°C for 5 min to aid Matrigel polymerization, and the droplets were then covered with spheroid-forming media. Spheroid colonies were formed by culturing the LCPs with basal DMEM/F12 medium supplemented with GlutaMAX, 2% B27, 1% N2, ampicillin (50 μg/ml), basic FGF (bFGF), epidermal growth factor (EGF), and insulin-like growth factor (IGF) (50 ng/ml each), the antioxidant 2-phospho-L-ascorbic acid (pVc; 280 μM), the histone deacetylase (HDAC) inhibitor valproic acid (VPA; 1 mM), and the Gsk-3β inhibitor CHIR99021 (CHIR; 3 μM). The medium was changed every other day for 10 days. For the Fgf2 inhibition experiment, we omitted the bFGF from the spheroid-forming media but added the potent Fgf2 receptor inhibitor AZD4547 to block any exogenous Fgf2 signaling.

Differentiation of the LPC spheroids into inner ear organoids started at D11. MI and control MACS organoids were placed in a solution containing GSK3β inhibitor, CHIR (3 μM), and the Notch inhibitor LY411575 (LY, 10 μM) to induce differentiation for 2 weeks. We also conducted a Notch inhibition experiment in which we doubled the concentration of Ly in the differentiation media to 20 μM. Furthermore, we conducted an Shh activation experiment in which we added the smoothened receptor agonist purmorphamine (1 μM, 04-0009; Stemgent, Beltsville, MD, USA) on D11. CHIR was replaced with the Wnt/beta-catenin inhibitor IWP-2 (3 mM, 3533; Tocris, Bristol, UK) on D18.

### E. qRT-PCR and immunohistochemistry

To analyze the expression of inner ear progenitor-related genes, 1 µg of RNA was collected from the MI and MACS spheroids at D10 using GeneAll Hybrid-R (GeneAll Biotechnology, Songpa-gu, Korea) according to the manufacturer's instructions. The forward and reverse primers (Oligomer; Bioneer) used to synthesize cDNA are listed in Supplementary Table S9. The qRT-PCR details are summarized in Supplementary Table S10.

Individual organoids were isolated and fixed with 2% paraformaldehyde for immunostaining. The organoids were then blocked with 5% normal goat serum and 0.3% Triton X-100 in PBS for 1 h at room temperature, followed by overnight incubation at 4°C with primary antibodies. Then, the slides were washed and incubated for 60 min at 37°C in appropriate secondary antibodies (listed in Supplementary Table S11). Finally, the samples were washed with PBS and mounted using VectaShield (Vector Laboratories, Newark, CA, USA) with or without DAPI. Stained cells were carefully examined under the Flow-View 3000 confocal microscope (Olympus, Tokyo, Japan).

### F. TEM and SEM

Ultra-morphological features of the D24 organoids were obtained via TEM and SEM. For TEM, the organoids were immediately fixed with 2.5% glutaraldehyde in phosphate buffer solution overnight. After being washed, the cultures were dehydrated in acetone and embedded in a mixture of Araldite and Epon 812 in flat rubber molds. The blocks were then mounted in an Ultracut Ultramicrotome (Leica, Wetzlar, Germany). Two micrometer semi-thin sections were cut with a glass knife and stained with 1% toluidine blue in 0.5% borax buffer. When a location of interest was found, 60-nm thin sections were cut with a diamond knife and collected on 300-mesh grids. After being stained with 3% uranyl acetate for 15 min and 1.5% lead citrate for 3 min, the samples were examined using a JEM-1400 transmission electron microscope (JEOL, Tokyo, Japan).

For SEM, organoids were washed for 30 minutes using cold Matrisperse Cell Recovery Solution to remove the Matrigel and fixed for another 30 minutes with 2.5% glutaraldehyde in 0.1 M sodium cacodylate buffer (pH 7.4). After fixation, the cells were washed three times (for 1 minute each) with 0.1 M sodium cacodylate buffer (pH 7.4), post-fixed for 10-15 minutes with 1% osmium tetroxide (OsO4) in the same sodium cacodylate buffer, and then washed. The fixed organoids were progressively dehydrated using a graded ethanol series, critical point-dried using hexamethyldisilazane (HMDS), and sputter-coated with gold/palladium (Au/Pd). The cells were then examined by field emission scanning electron microscopy (FE-SEM; Sigma 500; Zeiss, Oberkochen, Germany).

### G. Bulk RNA-seq and DEG analysis

RNA was purified using the RNeasy kit (Qiagen, Hilden, Germany). Approximately 200 ng of total RNA was used to construct the library for sequencing via the NEBNext® Ultra RNA Library Prep Kit for Illumina (New England Biolabs, Ipswich, MA, USA). Next, paired-end sequencing was performed using the NovaSeq 6000 sequencing instrument (Illumina, San Digo, CA, USA) according to the manufacturer's instructions, yielding 150-bp paired-end reads. The sequences were aligned to the *mus musculus* genome assembly GRCm39 using STAR version 2.7.8a. RSEM version 1.3.3. was used in combination with the STAR program. The default parameters were used in STAR and RSEM^72^-^75^. Differential expression analysis and enrichment tests were conducted using the DESeq2 v1.34.0 method to identify DEGs in the RNA-seq data. In this analysis, the expected counts were normalized, and thresholds of a log_2_ fold change (log_2_FC) ≥ 3 or ≤ -3 were applied, with a q-value < 0.01, to determine significant changes in gene expression^76^. The overall expression pattern was visualized in scatter, moving average, and principal component analysis plots generated using the ggplot2 R package. A volcano plot of the significant genes and heatmap of DEGs in the bulk RNA-seq were created using the EnhancedVolcano tool (available at https://github.com/kevinblighe/EnhancedVolcano) and hclust2 package v3.6.2 (available at https://github.com/SegataLab/hclust2), respectively. Functional classification analysis of the DEGs from the bulk RNA-seq was performed using the Metascape (https://metascape.org/) tool. This enabled us to predict the biological pathways that are statistically significantly related to the identified gene set 77 (we used an enrichment factor of 3 and a p-value threshold of 0.01 for filtering purposes).

### H. Single-cell RNA sequencing of organoids at differentiation day 20

For droplet-encapsulation scRNA-seq, > 5,000 cells were collected for analysis. A sequencing library was prepared using the Chromium Single Cell Reagent Kit v2 according to the manufacturer's protocol (10x Genomics, Pleasanton, CA, USA). The cell suspension was diluted to 500 cells per mL and quickly processed using the high-throughput Gemcode platform from 10x Genomics with v3 chemistry reagents. This system captured each cell in a droplet alongside a gel bead containing oligo(dT) primers, a unique cell barcode, and unique molecular identifiers (UMIs) within the lysis buffer. After that, the transcriptomes were converted to cDNA through reverse transcription. RT-PCR amplified the cDNA, and libraries were prepared from the 3' ends according to the manufacturer's protocol. The generated scRNA-seq libraries were sequenced on the NovaSeq 6000 Illumina platform using a high-output flow cell. We obtained sufficient paired-end reads to generate ~100-150 million fragments. After sequencing, cell barcodes, and UMIs were used to demultiplex the source cells and mRNA transcripts from the PCR-amplified cDNA.

For the scRNA-seq analysis, raw sequencing data for genes detected in > 60% of the cells were processed using 10x Genomics Cell Ranger software (v6.1.2). The Seurat R package (v3.1.3) was employed to analyze gene expression data from droplet-based sequencing experiments, including variable gene selection, dimension reduction, and clustering. All genes expressed in > 1% of cells, along with cells expressing over 500 genes with < 10% mitochondrial genes, were retained for differential expression analysis. Cluster type specificity scores were defined for each gene and cluster. Correlations among shared marker genes were examined using cell clusters of organoids as reference points. Differential gene expression analysis of all cell types in the dataset was performed, and marker genes were identified based on expression differences and statistical similarities (cut-off: log2FC ≥ 2, ≤ -2, and p-value < 0.01) via the Seurat package. This allowed us to focus on the genes that were most likely to play a role in HC development. UMAP plots and expression landscapes of key cellular markers critical to HC development were visualized using Loupe software (10x Genomics). Heatmaps displaying the expression patterns of cellular markers, categorized by cell type as identified in previous studies, were generated using the ggplot2 package in R. Additionally, functional classification and gene network analysis of cluster 11, identified as an inner hair-like cell type, were conducted using STRING analysis.

### I. SGN isolation and assembloids co-culture

To generate the IEO and SGN assembloids, spiral ganglion neurons were isolated from P1 wild-type C57BL/c6 mice pups. Dissection was done similar to the LPC isolation and the modiolus was harvested from the cochlea. For morphological assessment of the assembloids, the whole modiolus was explanted beside D24 Shh-treated EIO and allowed to integrate for 14 more days. For the MEA analysis, the modiolus were treated with collagenase at 37°C for 1 hour, followed by a dissociation process to obtain single cells. The isolated SGNs were then seeded at a cell density of 10^4 cells/well or together with D24 Shh-treated EIO cultured on 24-well MEA plates. The assembloids were co-cultured in the same medium composition as the organoid culture and incubated at 37 °C with 5% CO2 and 90% humidity. Media changes were done every other day.

### J. Microelectrode array (MEA)

The MEA plate was coated with a 0.1% PEI solution the day before cells were seeded. After coating, the IEO, SGNs, assembloid or cochlea explant were each seeded in a Matrigel dome onto the MEA plate. The spontaneous neural activities of organoids were recorded using the Maestro Edge™ platform running Axion software (AxIS version, Axion BioSystems, Atlanta, GA). The cells were maintained at 37°C under 5% CO2, controlled by the Maestro instrument. Organoid electrical activities were recorded for 20 min. The raw data were acquired using AxIS by Navigator at a sampling frequency of 12.5 kHz, and digitally filtered through a Kaiser Window with a high pass of 0.2 kHz and a low pass of 3 kHz.

### K. Statistical analysis

The datasets were analyzed using Prism software 8.4.3 (GraphPad Software, La Jolla, CA, USA; RRID:SCR_002798). The stereocilia length and FM1-43-positive cell counts were compared between the MI and MACS groups using two-tailed Mann-Whitney *U* tests (nonparametric) and unpaired nonparametric *t*-tests. To analyze multiple treatment groups for MACS, a nonparametric one-way analysis of variance (ANOVA) was performed, and Tukey's multiple comparison test was used as a post hoc test. P-values < 0.05 were considered to indicate significance.

## Supplementary Material

Supplementary figures.

Supplementary tables.

Supplementary video 1.

Supplementary video 2.

## Abbreviations

Brn3c: Brain-specific homeobox/POU domain protein 3C
CtBP2: C-terminal binding protein 2
D: Day
DEG: Differentially expressed genes
ESCs: Embryonic stem cells
EpCAM: Epithelial cell adhesion molecule
Espin: Autosomal recessive deafness type 36 protein
FACS: Fluorescence-activated cell sorting
F-Actin: Filamentous actin
FM1-43: N-(3-triethylammoniumpropyl)-4-(4-(dibutylamino)styryl) pyridinium dibromide
Gata3: GATA binding protein 3
GFP: Green fluorescent protein
GO: Gene ontology
HCs: Hair cells
NF-H: Neurofilament heavy chain
IEO: Inner ear organoids
IF: Immunofluorescence
Lgr5: Leucine-rich repeat-containing G-protein-coupled receptor 5
Lmo7: LIM domain only protein 7
LPCs: Lgr5-positive cells
Lrba: Lipopolysaccharide-responsive and beige-like anchor protein
MACS: Magnetic-activated cell sorting
MEA: Microelectrode array
MET: Mechanotransduction
MI: Manual isolation
Myo7a: Myosin VIIa
Nr2f1: Nuclear receptor subfamily 2, group F, member 1 antibody
Oct4: Octamer-binding transcription factor 4
P: Postnatal day
Pax2: Paired box gene 2
Pax8: Paired box gene 8
PCA: Principal component analysis
PCR: Polymerase chain reaction
Pou4f3: POU domain, class 4, transcription factor 3
PSD95: Postsynaptic density 95
Pvalb: Parvalbumin
RNA: Ribonucleic acid
RNA-seq.: RNA sequencing
scRNA-seq: Single cell RNA sequencing
SEM: Scanning electron microscopy
SGN: Spiral ganglion neuron
Shh: Sonic Hedgehog
Sox2: Sex determining region Y-box 2
t-SNE: t-Distributed stochastic neighbor embedding
TEM: Transmission electron microscopy
3D: Three-dimensional
Tuba4a: Acetylated alpha-Tubulin
UMIs: Unique molecular identifiers
UMAP: Uniform manifold approximation and projection
