# Supplementary material for: Shh agonist enhances maturation in homotypic Lgr5-positive inner ear organoids
Source: Theranostics. 2025 Apr 13;15(12):5543–65. doi: 10.7150/thno.107345 (PMC12068299; doi:10.7150/thno.107345)

Shh agonist enhances maturation in homotypic Lgr5-positive inner ear organoids

Nathaniel T Carpena [1†], So-Young Chang [2†], Seyoung Mun [3†], Kyung Wook Kim [4], Hyun C Yoon [5], Phil-Sang Chung [2, 6], Ji-Hun Mo [6], Jin-Chul Ahn [2, 7], Ji On Park[7], Kyudong Han [8], Ji Eun Choi [6], Jae Yun Jung [6], and Min Young Lee[2, 6, 7*]

[1] Department of Medical Laser, Graduate School of Medicine, Dankook University, Cheonan 31116, Republic of Korea

[2] Beckman Laser Institute Korea, Dankook University, Cheonan 31116, Republic of Korea

[3] Department of Academy-Industry Cooperation, Dankook University, Cheonan 31116, Republic of Korea

[4] Department of Orthopaedic Surgery, Dankook University Hospital, Dankook University College of Medicine, Cheonan 31116, Korea.

[5] Department of Molecular Science & Technology, Ajou University, Suwon, 443749, Republic of Korea

[6] Department of Otolaryngology-Head & Neck Surgery, College of Medicine, Dankook University, Cheonan 31116, Republic of Korea

[7] Medical Laser Research Center, Dankook University, Cheonan 31116, Republic of Korea

[8] Department of Microbiology, College of Science & Technology, Dankook University, Cheonan 31116, Republic of Korea

[†] These three authors contributed equally as co-first authors.

*** Corresponding Author: Min Young Lee**

Department of Otorhinolaryngology-Head & Neck Surgery, College of Medicine, Dankook University, 119, Dandae-ro, Cheonan-si, Chungnam, 31116, Republic of Korea

E-mail: eyeglass210@gmail.com

Supplemental Figure 1. Manual isolation technique: (A) Illustration of technique; (B) Experimental images

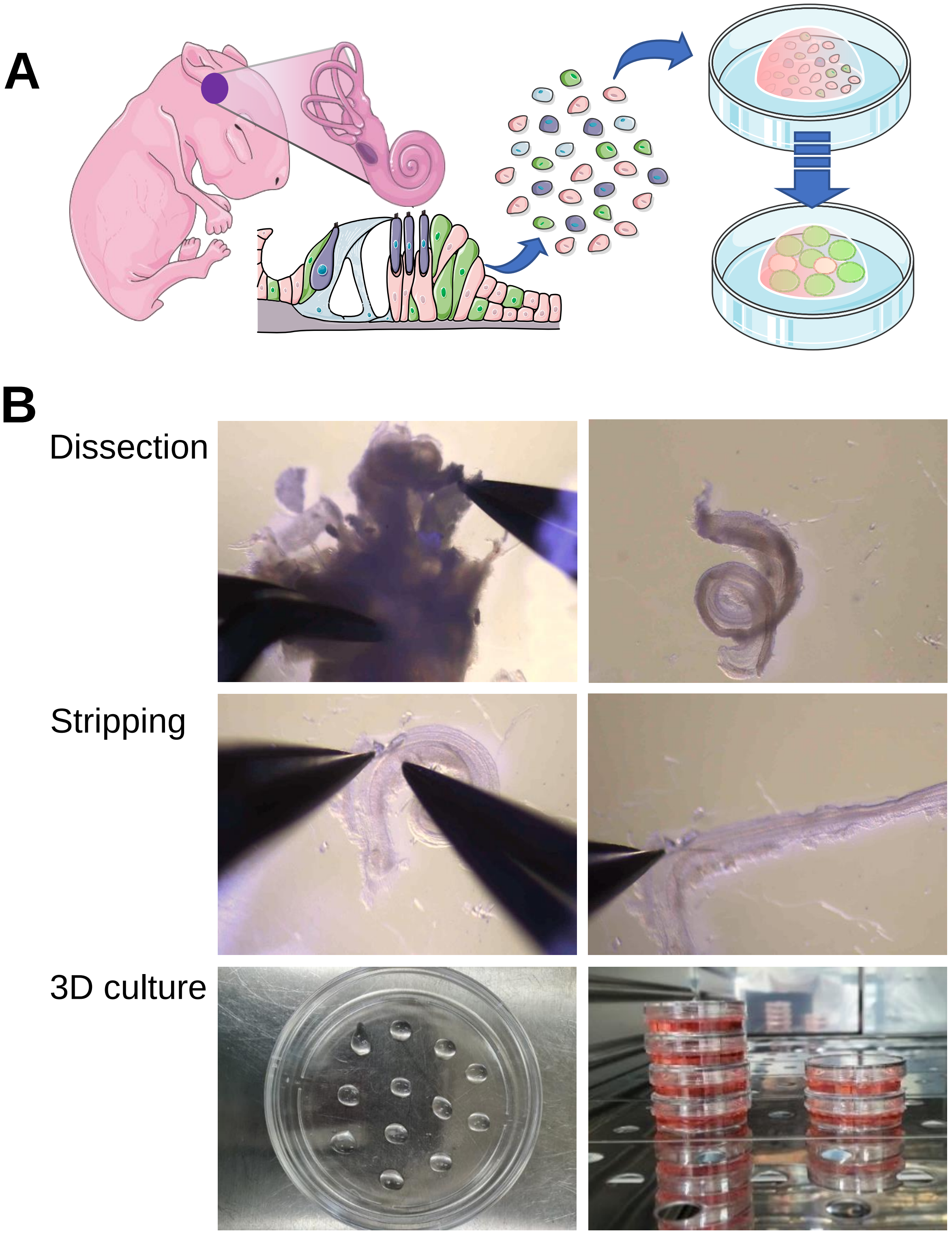

Supplemental Figure 2. MACS isolation technique (A to C: serial images)

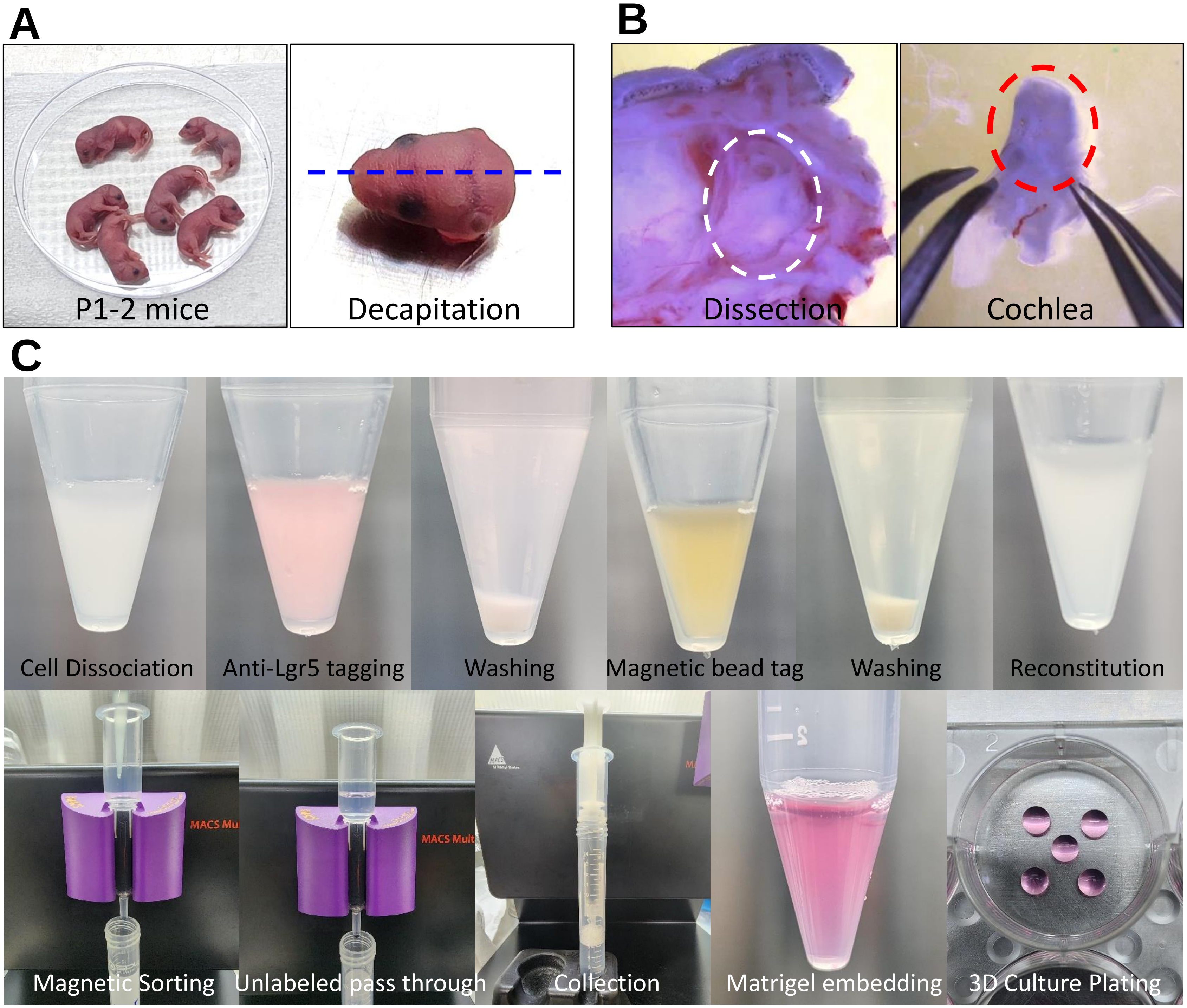

Supplemental Figure 3. Characteristics of MACS sorted LGR5-positive cells at D7

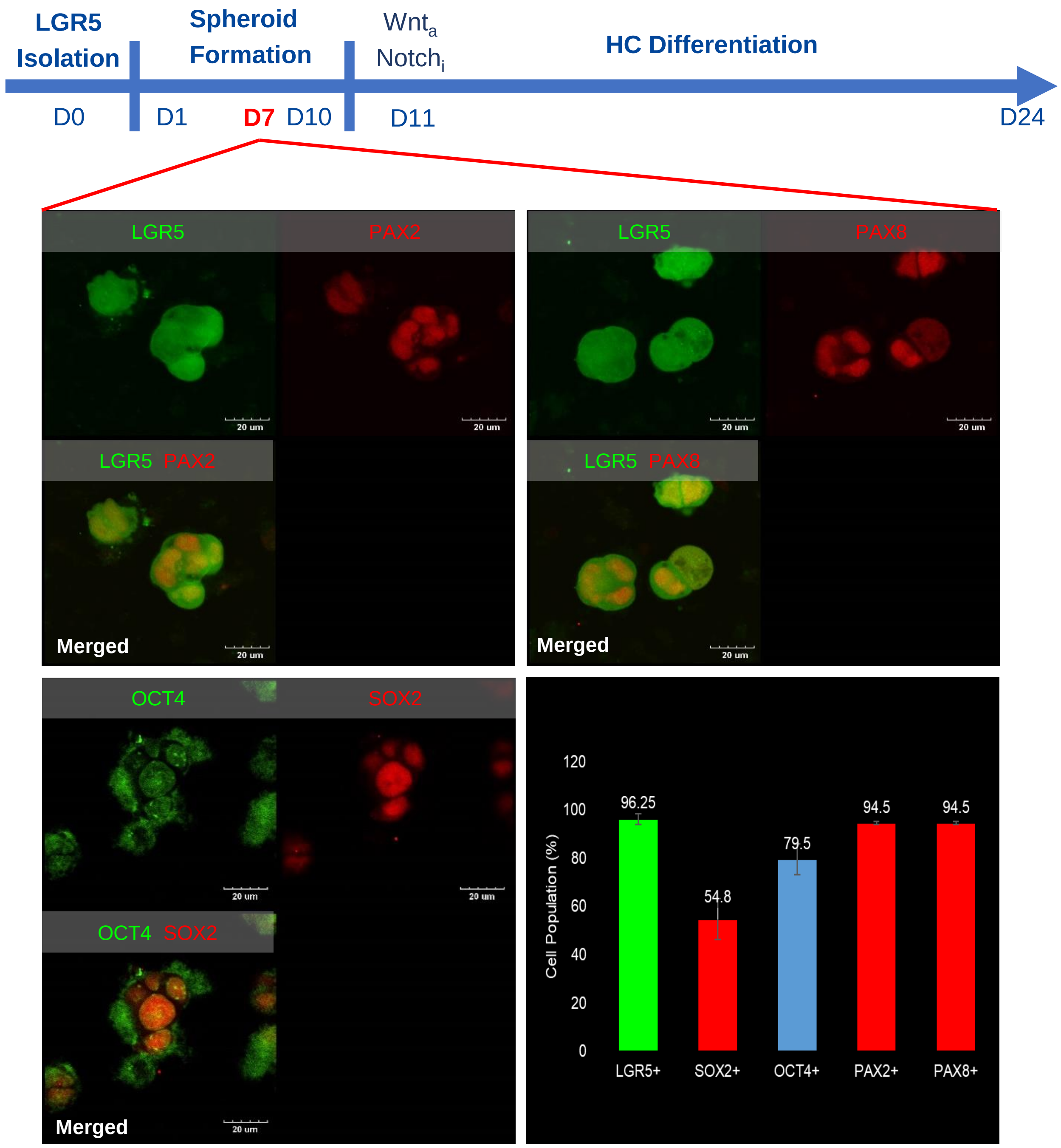

Supplemental Figure 4. Organoid generation from cells by Manual isolation technique (A) light microscopic images; (B) Epifluorescence analysis at D10 ; (C) Epifluorescence analysis at D30

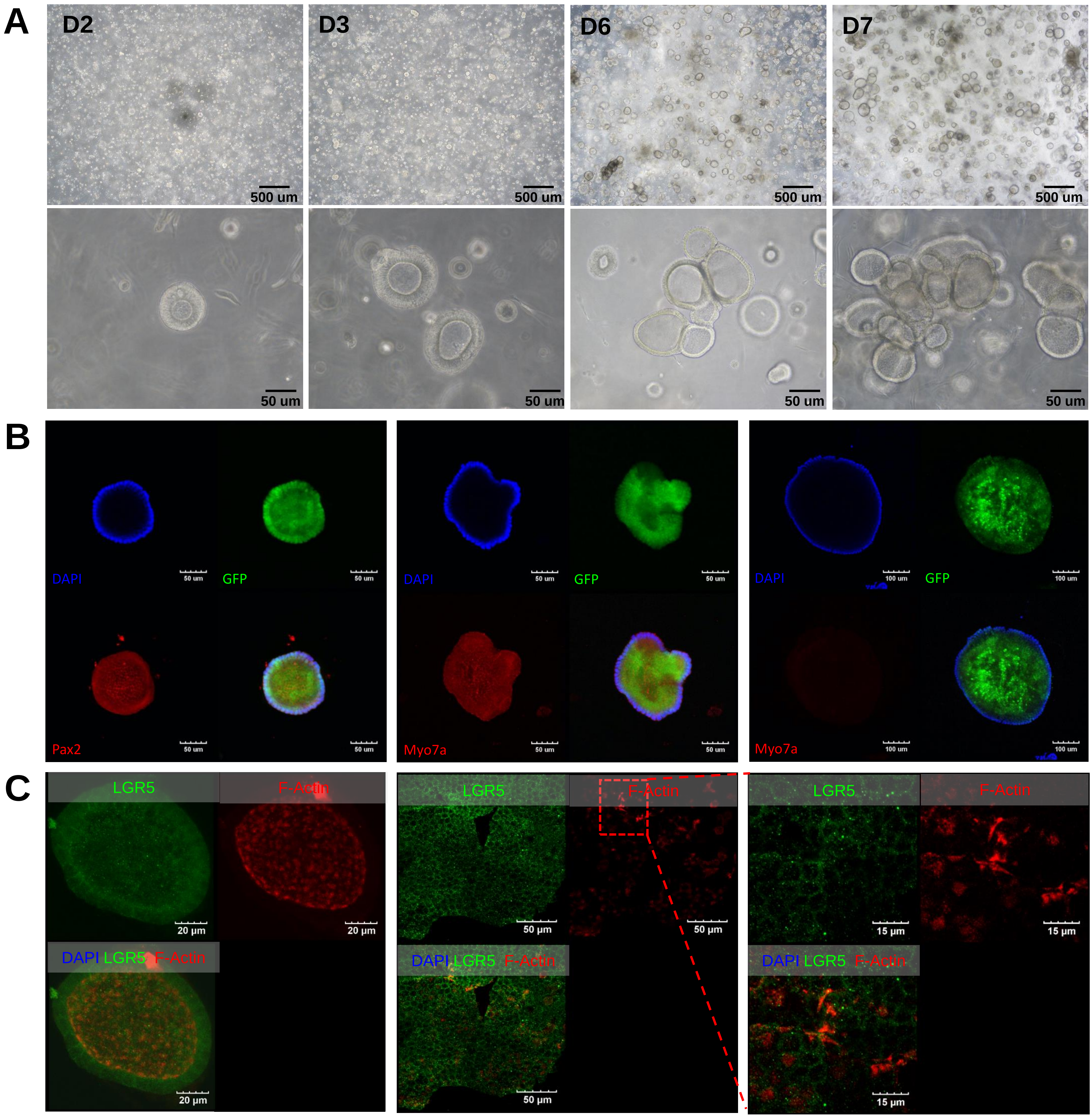

Supplemental Figure 5. Characteristics of organoid generated from MACS isolation LGR5 positive cells (A) Myo7a cell counts at D24, (B) Epifluorescence analysis.

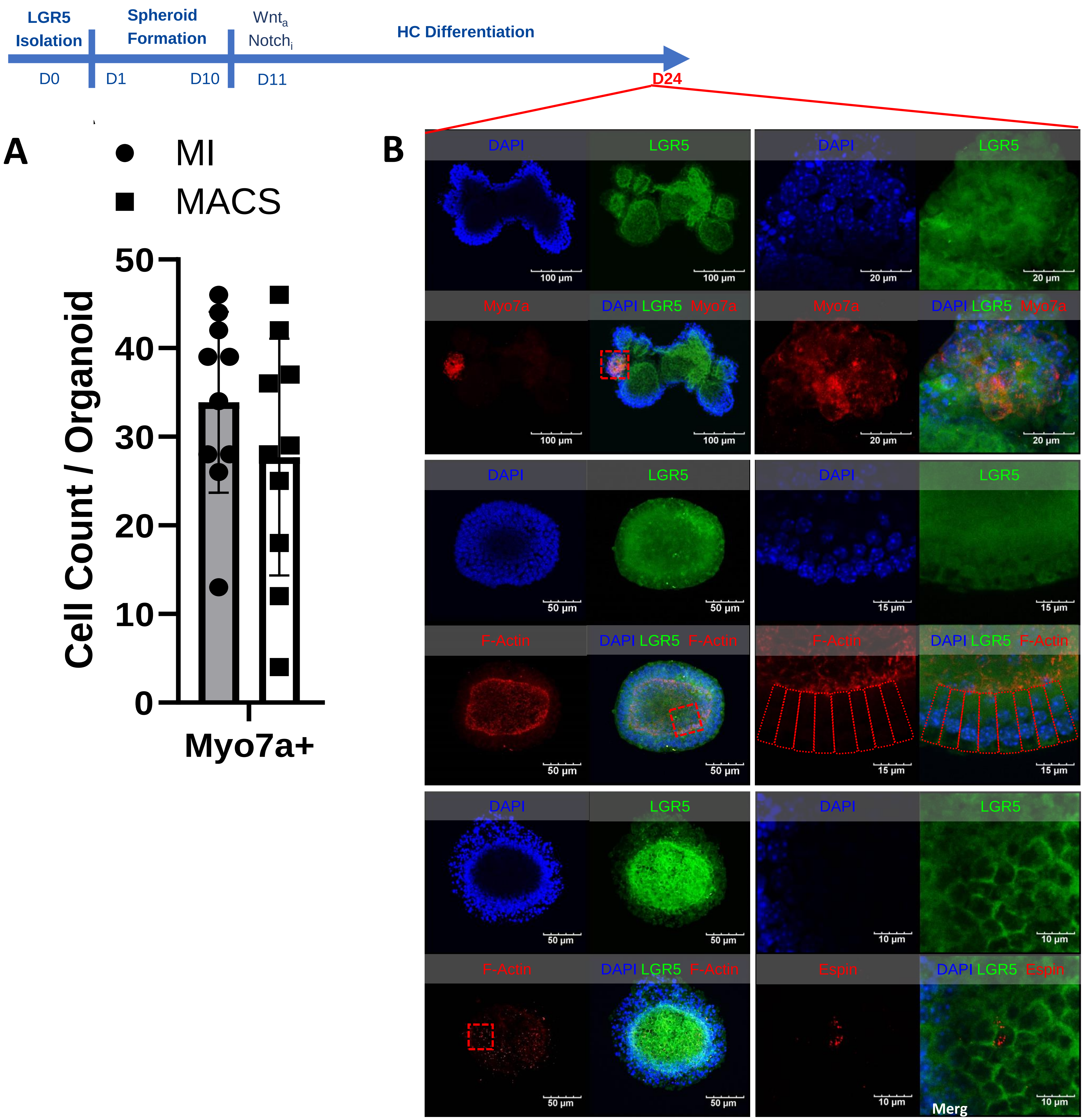

Supplemental Figure 6. Hair cell-associated gene expression between organoids from MACS and Manual isolation at DIV10

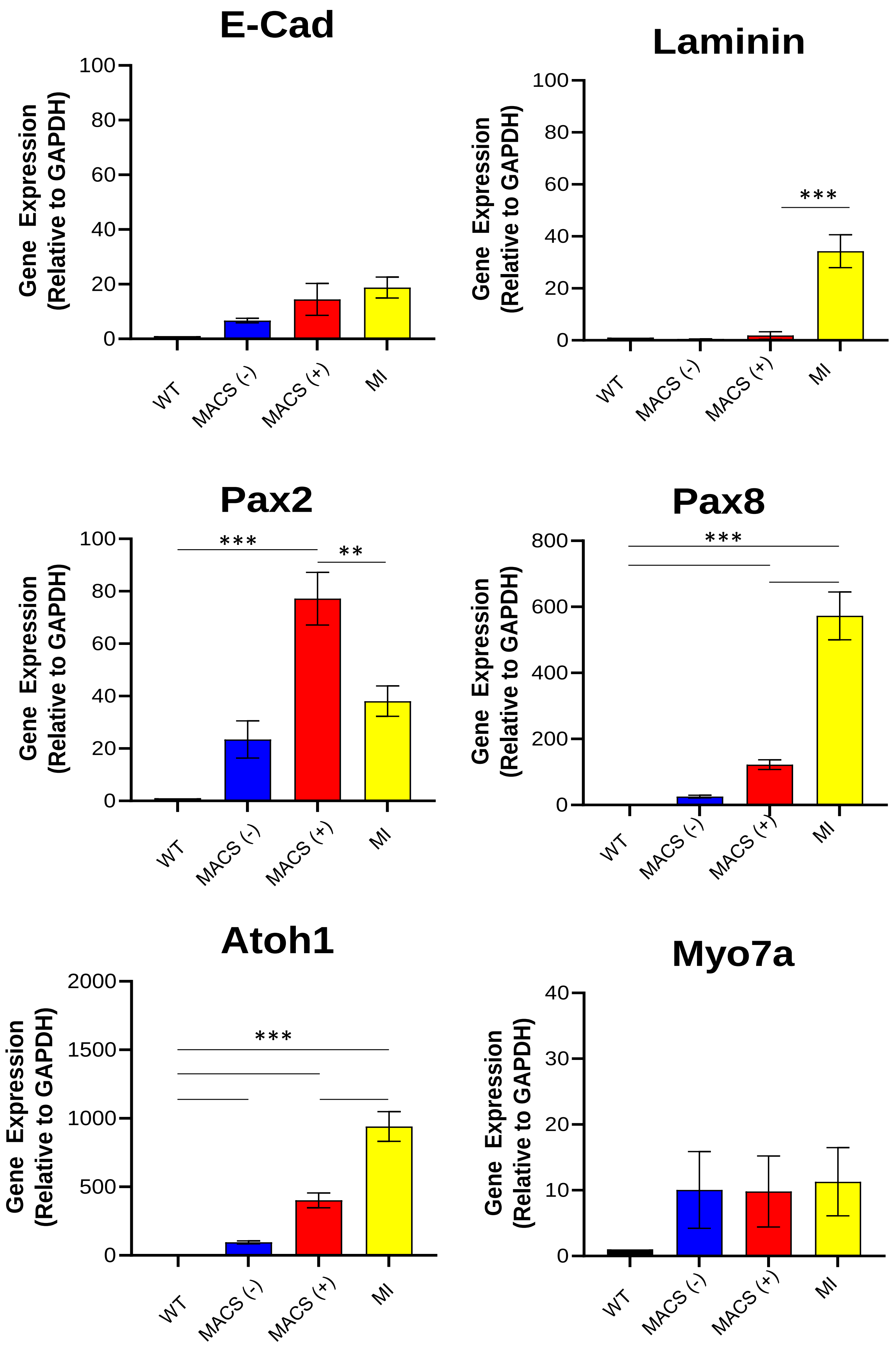

Supplemental Figure 7. RNA sequencing comparison of MI and MACS group at D10. (A,B) Statistical validation of RNA sequencing. (C,D) Gene distribution pattern difference of two group. (E) Gene Ontology regarding the differential gene expression between two group (DN: higher expression in MI group).

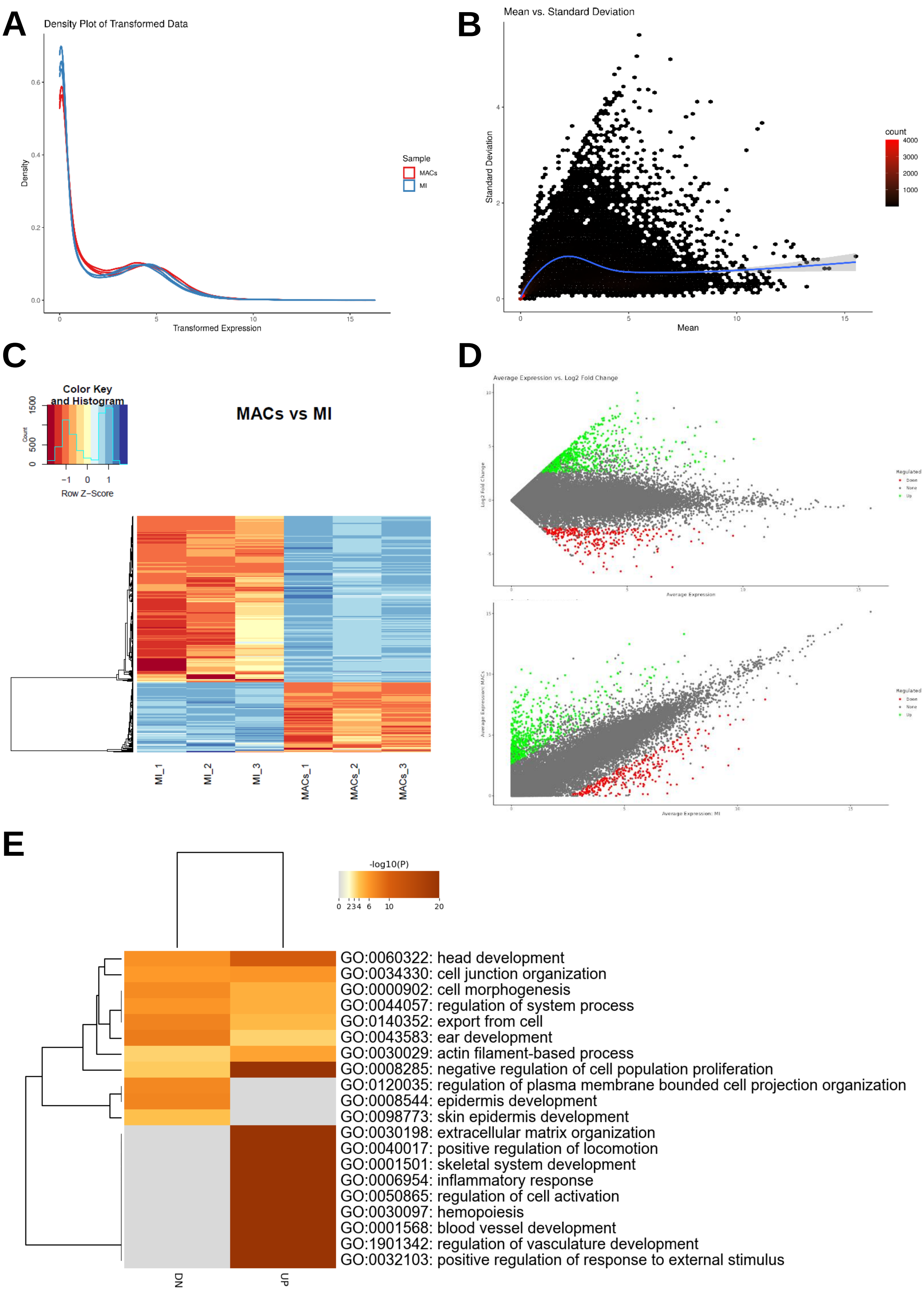

Supplemental Figure 8. Modifications on the HC differentiation protocol of MACS Lgr5-positive cells (A) Experimental timeline of 5 groups, (B) Representative light microscopic images of 5 groups at D2 and D10, (C) Spheroid sizes at D5 and D10.

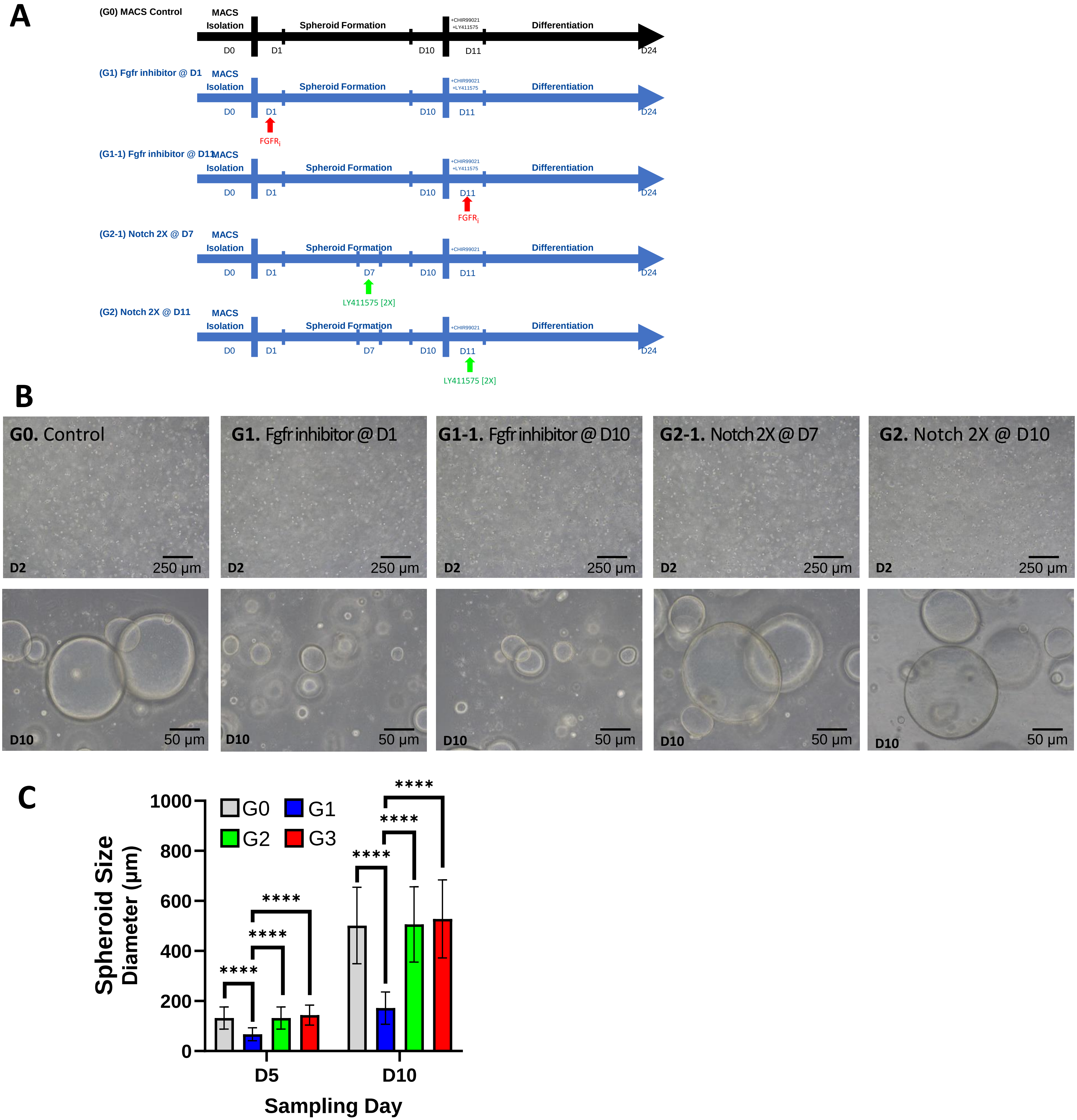

Supplemental Figure 9. HC morphology or organoids from different MACS experimental groups at D24

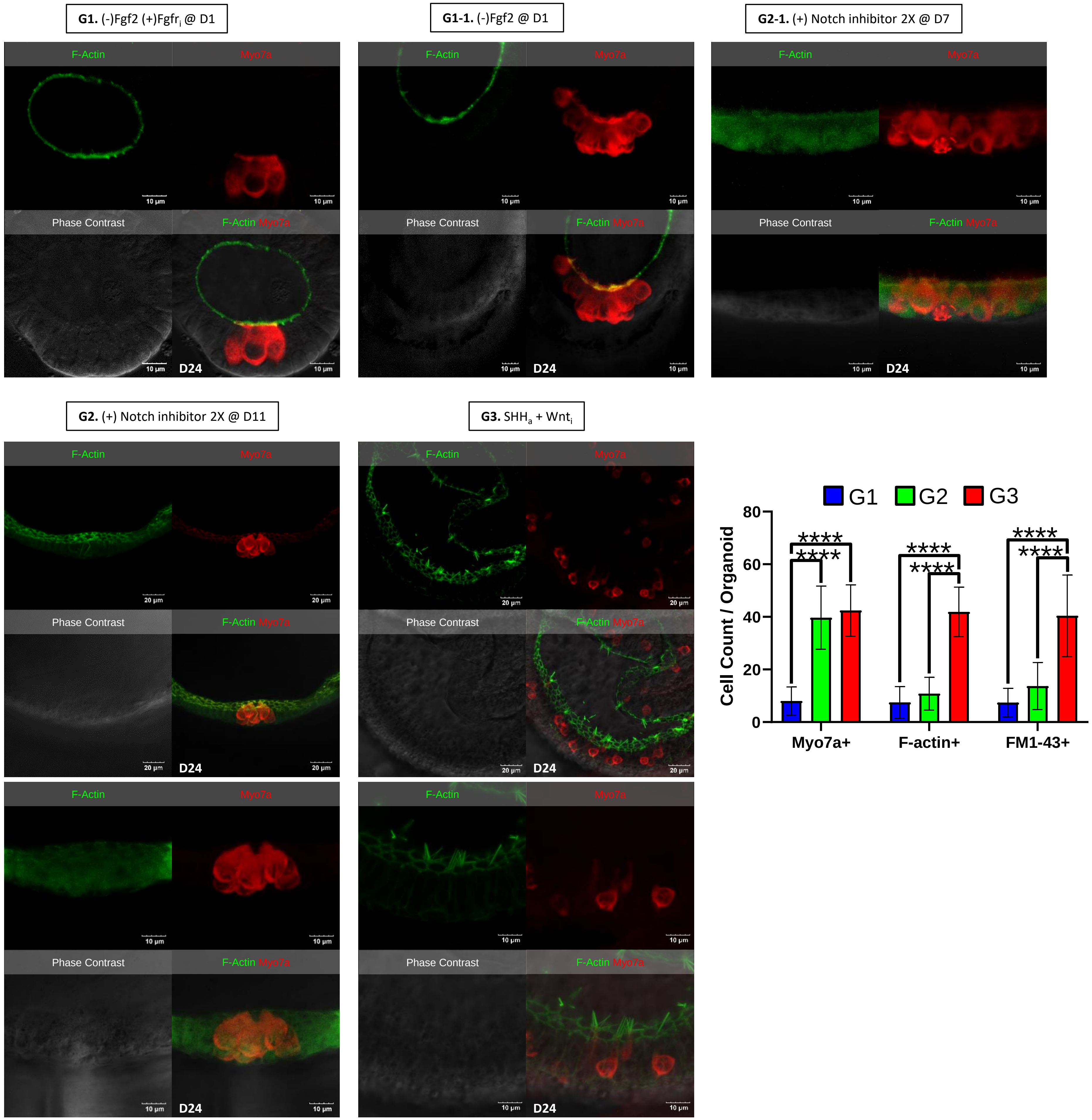

Supplemental Figure 10. Epifluorescence analysis of Lrba expression in the subapical region of hair cells

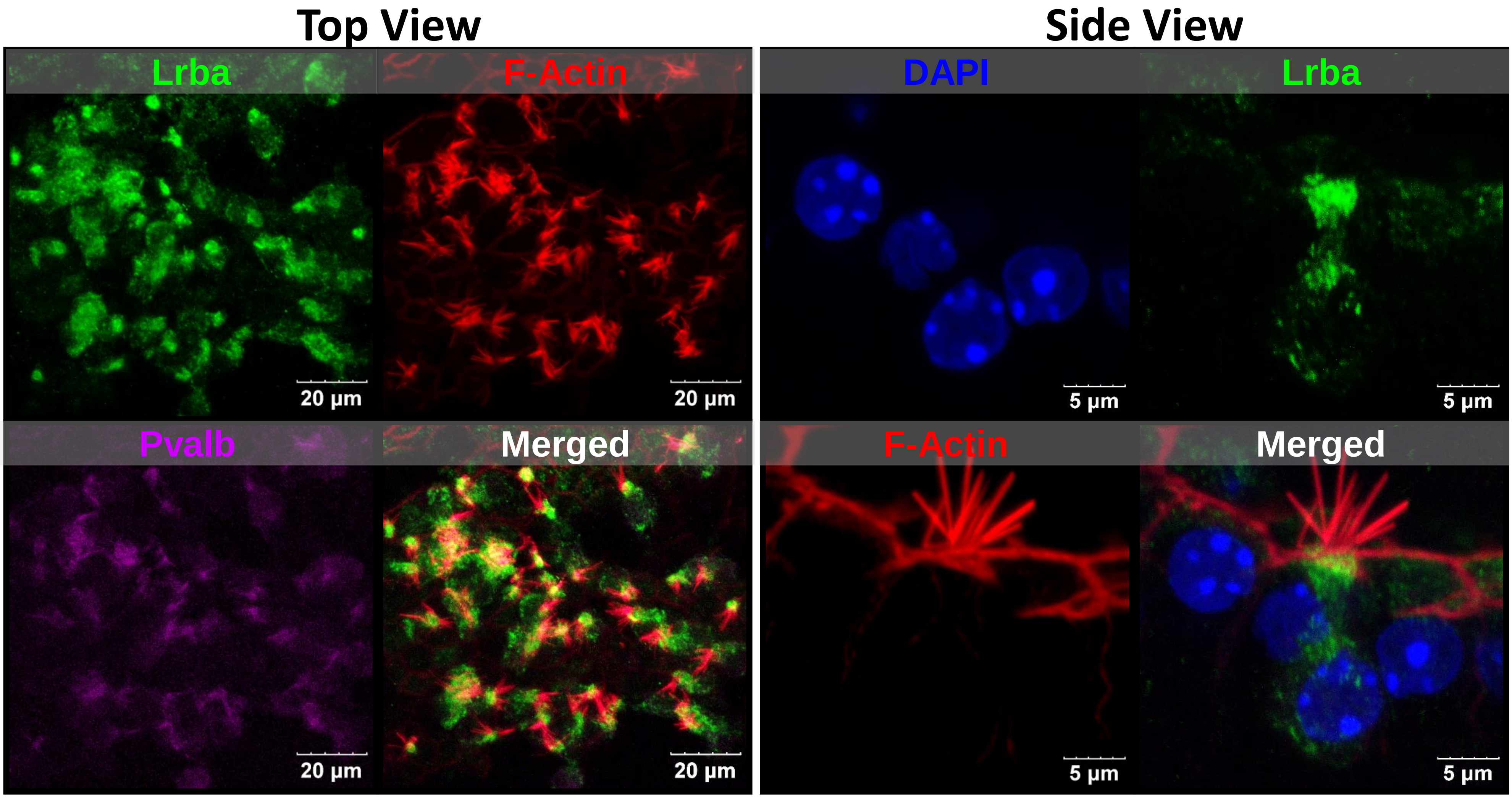

Supplemental Figure 11. Epifluorescence analysis of kinocilium marker, acetylated alpha tubulin (TUBA4A)

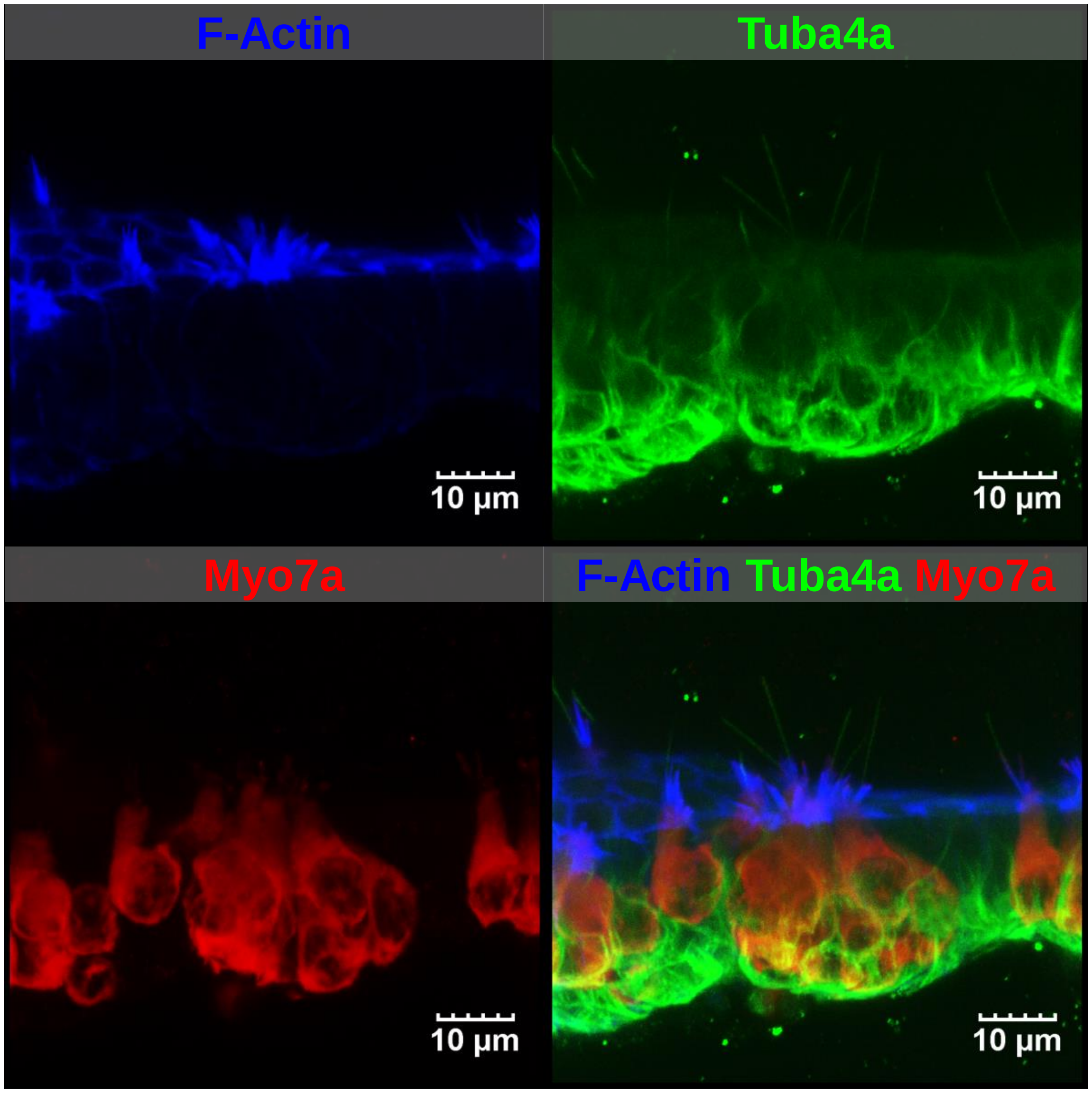

Supplemental Figure 12. TEM imaging of the stereocilia of differentiated HC. Ultrastructural imaging of hair cell stereocilia from LPC-derived IEO that differentiated using modified tratments. A. MACS Control, B. Fgfr inhibited at D1, C. Fgfr inhibited at D10, D. Notch Inhibition 2X at D10

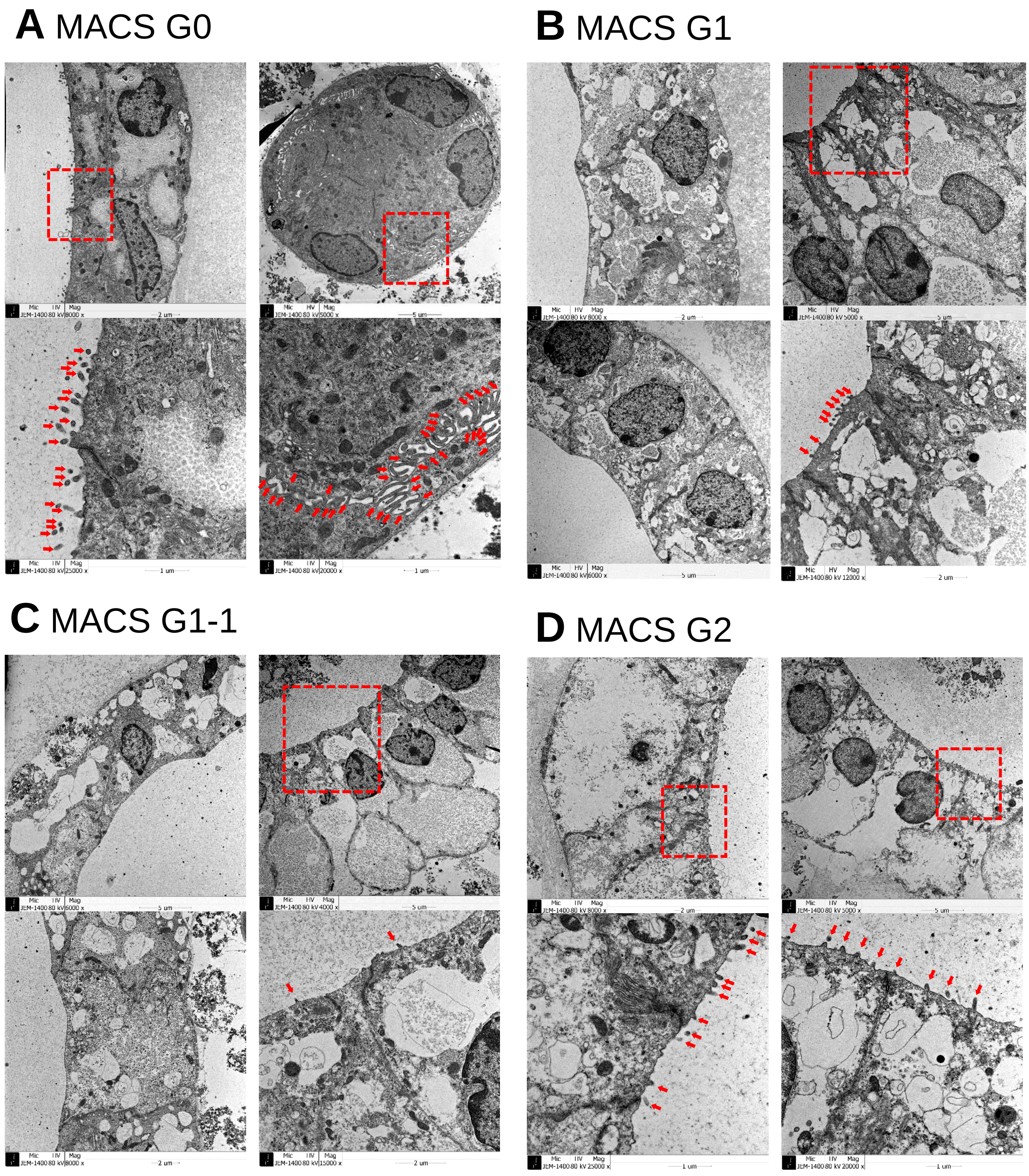

Supplemental Figure 13. RNA sequencing comparison of (G0: MACS_CL) MAC control and (G3: MACS_CL_PI) MACS SHH group at day 24. (A,B) Statistical validation of RNA sequencing. (C) Gene Ontology regarding the differential gene expression between two group. (D) Heatmap for differential gene expressions of postnatal hair cell genes (cite) between two group.

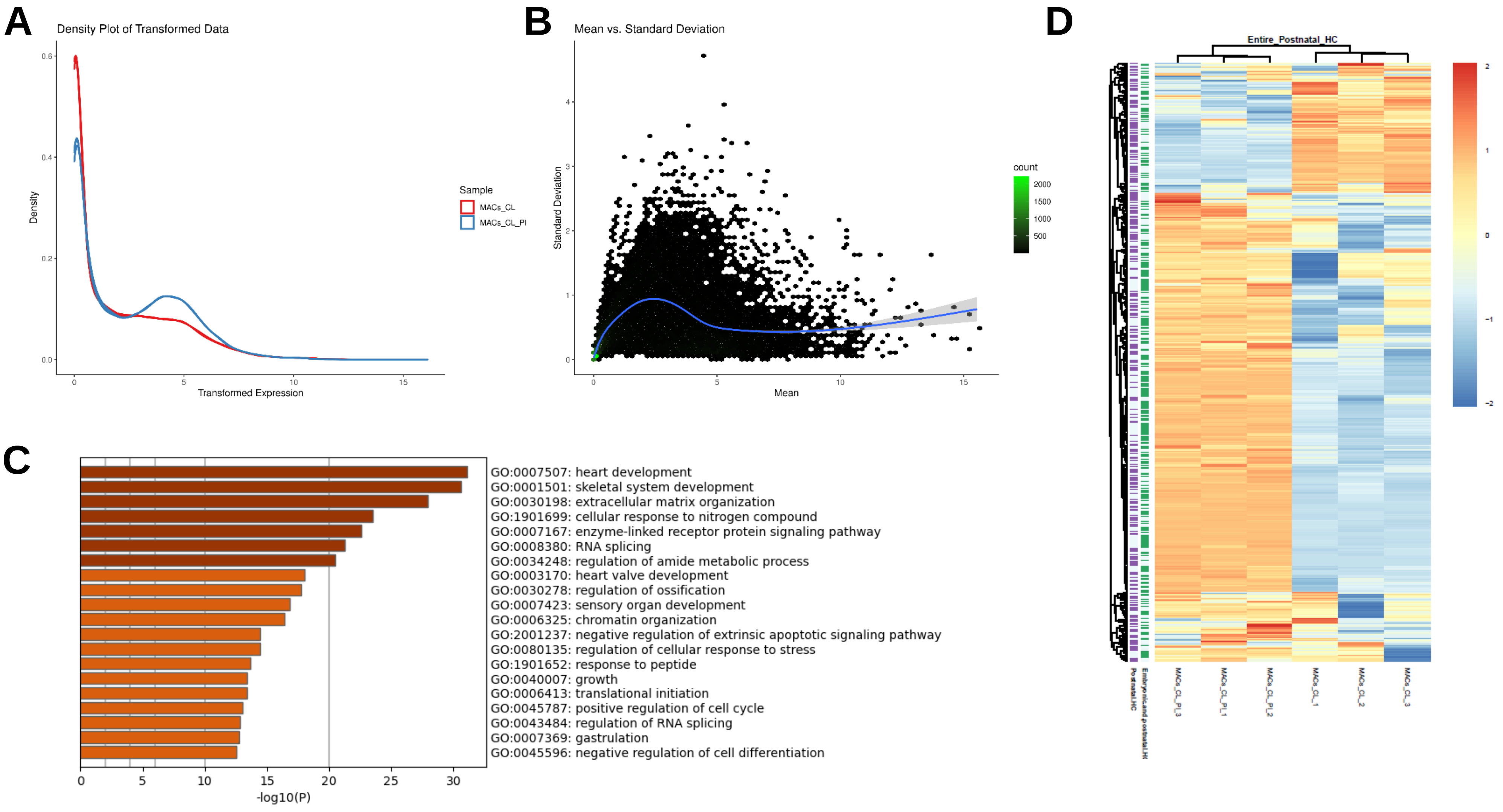

Supplemental Figure 14. Supplementary information for Single Cell (sc) RNA-seq

(A) Statistics summary of Scatter plot and percentage bar plot for filtering criteria and remaining cells. Dead cells and doublets were filtered out. (B) Heat map for postnatal hair cell markers suggested in previous studies. (C) Gene expression comparison heatmap for other cell types associated with the organ of Corti (D) Heatmap comparing expression of IHC and OHC cell markers using both bulk RNA-seq and scRNA-seq data. (B-D) The color intensity indicates higher gene expression levels in the plots. (E) Functional classification of up-regulated genes, identified from five clusters using scRNA-seq. Red boxes were added to emphasize the relevance of cilium development or stereocilia in HC clusters. The scale bars indicate the significant inclusion of genes in each cluster.

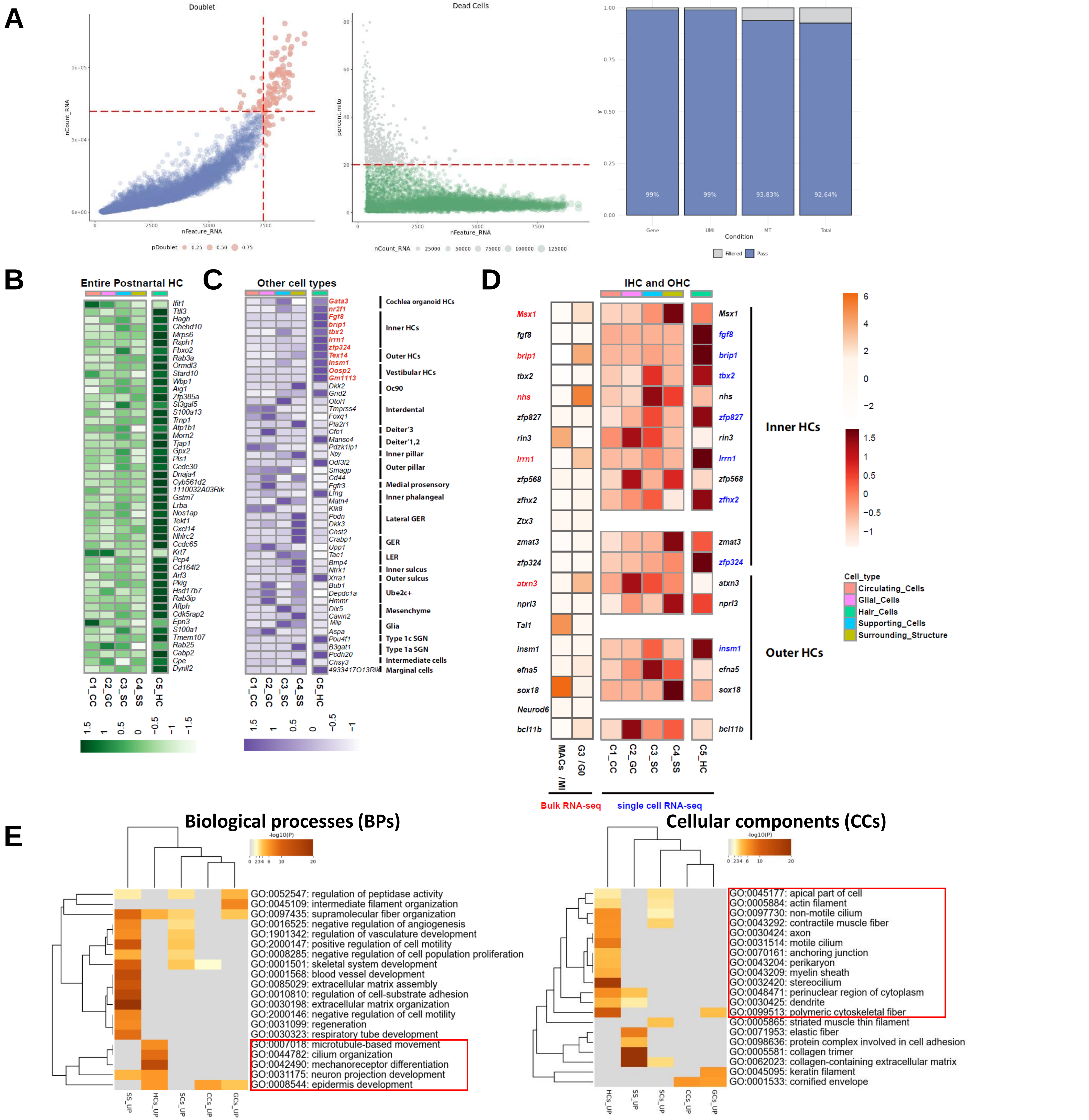

Supplement: Supplementary file 1 — Supplementary figures. [file thnov15p5543s1.pdf]
